# A non-canonical fungal peroxisome PTS-1 signal, SYM, and its evolutionary aspects

**DOI:** 10.1038/s41598-025-13871-x

**Published:** 2025-08-01

**Authors:** Judit Ámon, Suren Nemuuzaya, Kevin Alczheimer, Sándor Kocsubé, Zoltán Farkas, Gergő Svorenj, Attila Gácser, Chetna Tyagi, László Kozma-Bognár, Zsuzsanna Hamari

**Affiliations:** 1 Department of Biotechnology and Microbiology, University of Szeged Faculty of Science and Informatics, Szeged, Hungary; 2 Synthetic and Systems Biology Unit, Institute of Biochemistry, National Laboratory of Biotechnology HU-6726, HUN-REN Biological Research Centre, Szeged, HU-6726 Hungary; 3 HCEMM-USZ Fungal Pathogens Research Group, Department of Microbiology, Faculty of Science and Informatics, University of Szeged, Szeged, Hungary; 4 Department of Genetics, University of Szeged Faculty of Science and Informatics, Szeged, Hungary; 5 Institute of Plant Biology, Biological Research Centre, Hungarian Research Network (HUN-REN), Szeged, Hungary

**Keywords:** Microbiology, Fungi

## Abstract

Proteins localized to peroxisomes, particularly those expressed under specific conditions or in low abundance, are often undetected by routine proteomics methods due to detection sensitivity limits. In silico identification and experimental validation of peroxisomal targeting signals (PTSs) offer a reliable alternative. We demonstrate that SYM, a non-canonical plant PTS-1 signal, functions similarly in *Aspergillus nidulans*, as GFP tagged with a SYM C-terminal tripeptide localizes to peroxisomes. One of two native *A. nidulans* proteins with C-terminal SYM tripeptide shows weak peroxisomal localization alongside cytoplasmic presence, indicating that only a subset of proteins with non-canonical signals access peroxisomes. *In silico* analysis of 1,010 fungal genomes identified diverse SYM-proteins with variable functions, suggesting that non-canonical PTS-1 signals may evolve spontaneously. Two-thirds of SYM-proteins are predicted to localize to specific intracellular compartments other than the peroxisome. We propose that despite their predicted localization, these proteins possessing SYM as a non-canonical peroxisomal signal might also have peroxisomal presence. Among SYM-proteins, pectinesterases, known plant pathogen virulence factors, were frequent. Notably, 25% of fungal pectinesterases harbor non-canonical PTS-1 signals, suggesting that partial peroxisomal localization of pectinesterases has evolved convergently. This suggests that partial peroxisomal localization may enhance protein functional flexibility, contributing to the organism’s adaptability.

**Supplementary Information:**

The online version contains supplementary material available at 10.1038/s41598-025-13871-x.

## Introduction

Since the pioneering works on peroxisomes^1^great efforts has been devoted to uncovering the full spectrum of biological processes occurring within these organelles and elucidating the mechanisms by which proteins are targeted to- and imported into peroxisomes (recently reviewed in^2^.

In eukaryotes, most peroxisomal proteins are targeted through two primary import pathways. These pathways recognize specific motifs: PTS-1, interacting with the Pex5 receptor, and PTS-2, interacting with the Pex7 receptor (reviewed in^3^. A typical PTS-1 motif consists of three amino acid (AA) residues at the carboxy-terminus of a protein, with a consensus motif of [A/C/H/K/N/P/S/T]-[H/K/N/Q/R/S]-[A/F/I/L/M/V]^4^). In contrast, the PTS-2 motif is composed of nine amino acids at the N-terminal region, with a consensus motif of [R/K]-[L/V/I/Q]-X-X-[L/V/I/H/Q]-[L/S/G/A/K]-X-[H/Q]-[L/A/F]^5^). Peroxisomal protein import also occurs through non-conventional mechanisms. For example, a PTS-3 signal is formed by distantly positioned amino acids creating a structural patch on the protein surface, enabling interaction with Pex receptors and subsequent import through the cognate PTS machinery^6^. Additionally, there are true PTS signals, which are called cryptic because they cannot be inferred from the coding sequence. Instead, these signals are generated through processes such as alternative splicing or ribosomal read-through^7^. Finally, proteins lacking PTS signals can be imported into peroxisomes through piggy-back transport, where a PTS-containing protein physically interacts with a PTS-lacking protein, facilitating its co-import into the peroxisomes^8,9^.

In the era of omics and high throughput techniques, proteome analysis of isolated peroxisomes (e.g. *Arabidopsis thaliana* peroxisomal proteome research reviewed by^10^ and construction of and screening of Gfp-fusion protein libraries in easily transformable organisms (e.g. study of intracellular localizations of proteins covering 75% of the *Saccharomyces cerevisiae* proteome^11^ have outpaced traditional investigation approaches. However, these approaches fail to identify peroxisomal proteins (PPs) when they are present at sub-detectable levels or are expressed only under specific environmental conditions. As a result of these limitations, the detection of PTS signals, followed by experimental localization studies, remains a valuable method, particularly if the accuracy of *in* *silico* PTS recognition can be further improved.

The key bottleneck in *in silico* PTS detection lies in the accuracy of the applied PTS-prediction algorithms. The most advanced PTS-1 predictions for plants stem from comprehensive studies of the *A. thaliana* peroxisomal proteome, which uncovered a wide range of non-canonical PTS-1 signals^12–15^. Reumann et al. expanded the canonical PTS-1 ([A/S] [K/R] [I/M/L]) and the relaxed PTS-1 formula ([A/C/H/K/N/P/S/T]-[H/K/N/Q/R/S]-[A/F/I/L/M/V]) by incorporating the experimentally-validated non-canonical motifs. The resulting Reumann’s formula [A/S/(Q/I/K/L/T/G/V/F/C/P)] [K/R/(A/D/Y/C/Q/P/F/T/E/G/H/M/L/N/S)] [I/M/L/(F/Y/V)] includes non-canonical residues in parentheses^15^. According to Reumann et al., *“a functional plant PTS-1 tripeptide must contain at least two canonical residues”*^15^. However, they also validated peroxisomal signals with one canonical and two non-canonical residues at their C-temini^16^. Furthermore, seven upstream residues adjacent to the C-terminal tripeptide were found to modulate interactions with Pex5 receptors, with kingdom-specific variations^17,18^ and these must be considered in *in silico* localization predictions. The PredPlantPTS1 web server (http://ppp.gobics.de/) utilizes the most developed PTS-1 describing formula and accounts for at least ten C-terminal residues, enhancing the precision of PTS-prediction^19^.

Reumann et al. demonstrated that proteins with non-canonical PTS-1 motifs exhibit weaker interaction with Pex5 receptor compared to those with canonical PTS-1 motifs, resulting in less efficient peroxisomal import^15^. Based on the functional analysis of non-canonical PTS-1 carrying PPs, they proposed several biological explanations for the weaker receptor binding properties of these proteins. These include: (i) the protein may be in the process of acquiring peroxisomal targeting, (ii) it may have dual (e.g. cytoplasma/peroxisome) localization, or (iii) it may require an extended period of cytoplasmic residence for proper maturation, with slower import into peroxisomes facilitating this process. The latter scenario was experimentally validated with the fungal peroxisomal catalase from *Hansenula polymorpha*, which has a canonical PTS-1 motif, SKI^20^. This SKI-carrying catalase showed an 8-fold lower affinity for the Pex5 receptor compared to a modified catalase with the most canonical PTS-1 motif, SKL. The lower affinity of the SKI motif led to a prolonged cytoplasmic residence time, allowing the catalase to fold properly before peroxisomal import. Replacement of the SKI motif to SKL resulted in peroxisomes populated by improperly folded, inactive catalases. This confirmed that the prolonged cytoplasmic residence provided by the SKI motif is essential for the proper folding and functional maturation of peroxisomal catalase prior its peroxisomal import^20^.

Detection of proteins with non-canonical PTS-1 motives in the peroxisomes are challenging, if not impossible, due to their low abundance in these organelles^7,15^. Thus, the failure to detect a non-canonical PTS-1 carrying protein in peroxisomes does not rule out the possibility that a small fraction of the protein is indeed present there.

Several peroxisomal proteins with non-canonical PTS-1 signals have been identified in isolated peroxisomes of *A. thaliana*. One of these is a short chain dehydrogenase (At3g01980) with a C-terminal SYM (Ser-Tyr_Met) motif^12^. The functionality of this SYM motif has been experimentally validated by C-terminal tagging of Gfp reporter protein with the SYM motif, demonstrating its ability to direct proteins into peroxisomes^12^.

In addition to their general functions, such as fatty acid β-oxidation, peroxide neutralization, detoxification, peroxisomes have also been implicated in specific biosynthetic and catabolic processes in *Aspergillus nidulans*, a metabolically versatile model organism among filamentous Ascomycota. These include a step in biotin biosynthesis catalyzed by 8-amino-7-oxononanoate synthase, BioF^21^; a reaction in nicotinic acid degradation catalyzed by 6-hydroxynicotinic acid monooxygenase, HxnX^22^; and steps of purine catabolism involving the enzymes UaZ, UaX, and UglA^23^. Additionally, certain steps in the biosynthesis of secondary metabolites also occur in peroxisomes, such as the reaction catalyzed by acyl-CoA:6-aminopenicillanic acid acyltransferase, AatA, during penicillin biosynthesis^24^.

In most reported cases, efficient targeting by non-canonical PTS-1 signals is attributed to upstream amino acid residues that enhance the interaction between the protein’s C-terminus and the TPR domain of the Pex5 receptor. Therefore, most known examples of non-canonical signals linked to peroxisomal localization are generally based on Gfp fusion experiments, where the fusion proteins show clearly detectable subcellular localization. One such case is the Gfp-AatA fusion protein, which showed strong peroxisomal targeting despite having a non-canonical C-terminal tripeptide (ANI, where the Asn (N) at position − 2 is not typical and considered a non-canonical residue)^24^. The importance of the seven upstream residues for Pex5 binding to non-canonical PTS-1 signals has been demonstrated experimentally in plants^17,18^ and observed in yeast as well. For instance, the non-canonical CKL signal on Idp3 (isocitrate dehydrogenase 3) mediates efficient peroxisomal import of Gfp-Idp3, whereas the cytoplasmic Idp2 (isocitrate dehydrogenase 2), when tagged with the same CKL motif, shows dual localization: a weak peroxisomal targeting alongside a predominant cytoplasmic localization^25^.

Systematic investigation of such weak or undetectable signals from fluorescent fusion proteins are lacking in the field. In most case, proteins are considered non-peroxisomal if no Gfp signal is detected in the peroxisomes. This limits the chances of identifying peroxisomal localization, especially since PTS-1 prediction tools often fail to recognize non-canonical signals. In the absence of a positive prediction, experimental validation of the localization is rarely pursued.

In this study, we emphasize the potential functional relevance of non-canonical motifs, even when fluorescent assays do not show clear peroxisomal localization. For this purpose, we examine the occurrence and targeting ability of a randomly selected, rare non-canonical signal known from plants, SYM^12^ in *A. nidulans*, a model filamentous fungi. By screening the *A. nidulans* protein database for proteins with the C-terminal tripeptide SYM (hereafter referred as SYM-proteins), we identified two candidates: AN1402 and AN5316. Neither these proteins nor the SYM-tagged GFP were predicted to localize to peroxisomes using the Reumann’s formula (via the PredPlantPTS1 web server) or other prediction tools (e.g. Yloc, WoLF PSORT II, DeepLoc 2.0). However, we hypothesized that the SYM motif functions as a *bona fide* non-canonical PTS-1 signal in fungi. In this work, we experimentally proved that the SYM motif indeed acts as a *bona fide* PTS-1 signal in *A. nidulans* and demonstrated partial peroxisomal localization of one of the *A. nidulans* SYM-proteins, AN5316. Furthermore, through *in silico* analysis of protein databases of 2,267 fungal species, we investigated the occurrence and distribution of SYM-proteins across the fungal kingdom. Our results support the hypotheses that non-canonical PTS-1 signals may arise spontaneously through point mutations^15^.

## Results

### SYM-tagged Gfp enters peroxisomes in the model fungus, *Aspergillus nidulans*

**Fig. 1:**
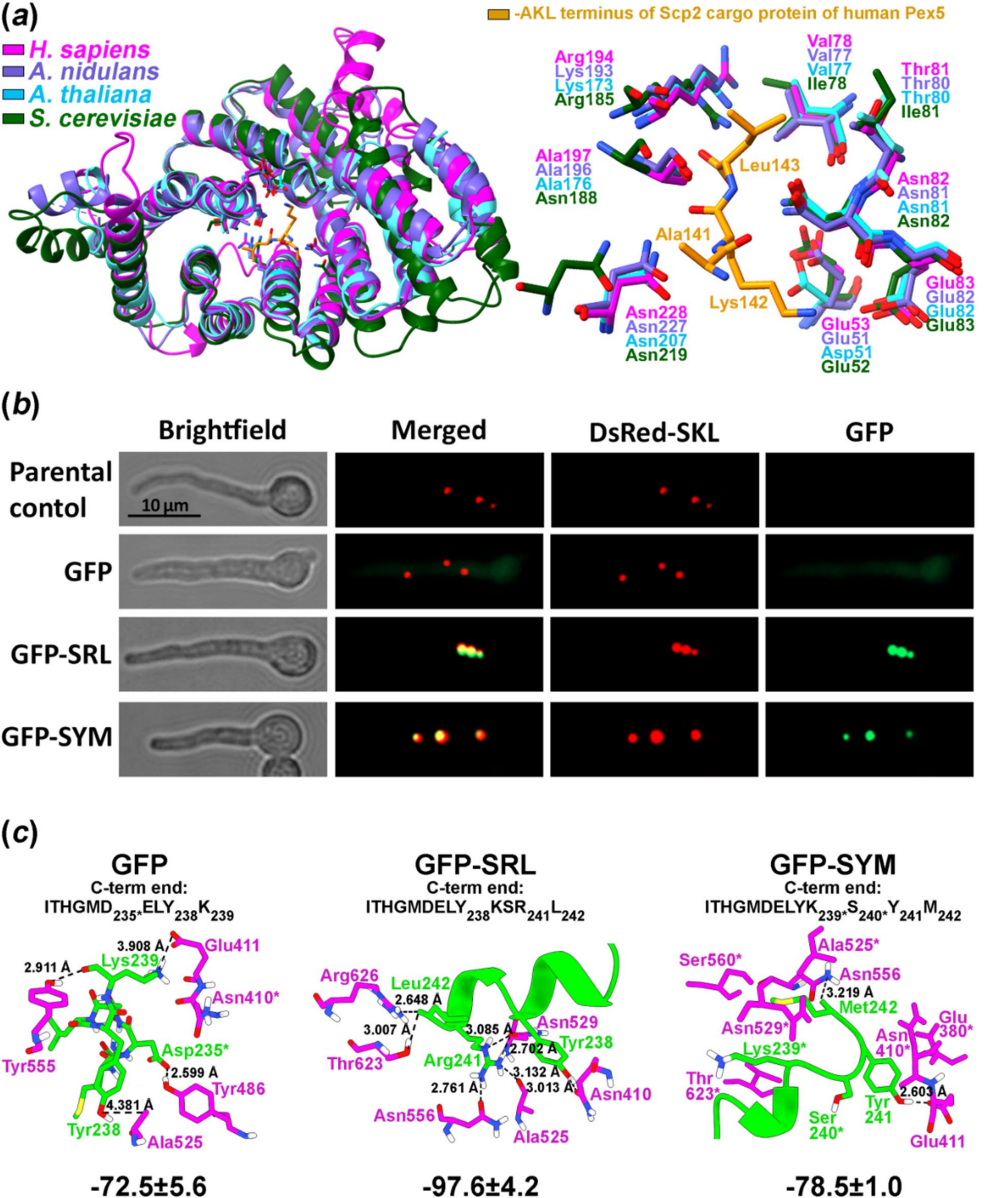
Analysis of SYM C-terminal tripeptide, a possible non-canonical PTS-1 signal in fungi. (**a**) Structural superposition of Pex5 homologs from *A. nidulans*, *A. thaliana* (Q9FMA3) and *S. cerevisiae* (YDR244W) with their closest known structural homolog, human Pex5 (left), and the AA side chains interacting with the AKL PTS-1 signal of a natural cargo protein, human Scp2 (sterol carrier protein 2 (PDB code: 2C0L) (right). Color codes are explained in the figure. (**b**) Intracellular localization of the SYM-tagged Gfp protein (Gfp-SYM) overlaps with the co-expressed SKL-tagged red fluorescence protein (DsRed-SKL), which marks peroxisomes^21,27^ (strain HZS.573). As controls, SRL-tagged Gfp and un-tagged Gfp were co-expressed with DsRed-SKL in strains HZS.952 and HZS.948, respectively. Fluorescence microscopy was performed using Zeiss filter sets 09 and 15 for DsRed and Gfp, respectively. Conidia were germinated for 6.5 h at 37 °C on coverslips submerged in MM followed by imaging. Scale bar: 10 μm. Gfp levels produced in the tested strains were determined by Western blot (Supplementary Fig. S2). (**c**) Interaction of *A. nidulans* Pex5 homolog PexE (magenta) with Gfp and PTS-1 tagged Gfps (Gfp-SRL and Gfp-SYM) (green). Hydrogen bonds are indicated with dashed lines, and distances between interacting atoms are given in Ångströms. Asterisks denote van der Waals or other secondary interactions. For each PexE-cargo complex, the sequence of the C-terminal 10–13 AAs is shown, with the interacting residues in the cargo indicated by subscripted positions. Below each complex, the binding energy (in kcal/mol) is shown, as calculated by HADDOCK server using the full-length sequences of PexE and the cargo proteins. Lower kcal/mol values indicate stronger interactions.

To understand the structural basis of PTS-1 recognition by Pex5 homologs and to assess how non-canonical targeting signals may be accomodated, we first compared the structures of peroxisomal import receptors across species. Comparative structural analysis revealed that PexE from *A. nidulans* (structure was generated by AlphaFold3 server) shares high structural similarity with Pex5 from both *A. thaliana* (Q9FMA3) and human (PDB code: 2C0L) (Fig. 1, Panel A). Given that the interaction between the TPR domain of human Pex5 and its canonical cargo protein Scp2 (sterol carrier protein 2, bearing an AKL PTS-1 motif), has been resolved by X-ray crystallography^26^ we examined the superposition of Pex5 homologs from *A. thaliana*, *A. nidulans*, and *S. cerevisiae* (YDR244W), focusing on the residues involved in AKL binding within the TPR domain cavity (Fig. 1a). The TPR domains of these Pex5 homologs aligned closely, with high, but not complete conservation of key side chains between *A. thaliana* and *A. nidulans* compared to human Pex5 at the AKL binding site (Fig. 1a). Subtle structural differences among Pex5 homologs from these three eukaryotic lineages likely explain the observed variation in predicted binding energies and interacting residues derived from docking simulations (HADDOCK server), summarized in Supplementary Fig. S1. We found that canonical PTS-1 tripeptides confer strong binding to all Pex5 homologs tested across species (Supplementary Fig. S1a). Loss of a single canonical residue reduced binding affinity but could be partially compensated by AAs upstream to the terminal tripeptide, which form alternative stabilizing interactions within the TPR cavity (Supplementary Fig. S1a and S1c). Loss of two canonical residues led to weaker interactions, unless substitutions introduced aromatic side chains (e.g., SYF, SYY), which may enhance binding via additional stabilizing upstream contacts (Supplementary Fig. S1a and S1c). In PTS-1 tagged Gfp constructs, Lys239 (at position − 4) and Tyr238 (at position − 5) corresponding to the last two C-terminal residues (positions − 1 and − 2) in the non-tagged Gfp protein (Supplementary Fig. S1b), emerged as key contributors to the stabilization of non-canonical signals. Finally, the concave shape of the TPR domain surface also influenced the binding conformation of the C-terminal targeting signals, as illustrated by the AKL motif’s interaction with *A. nidulans* PexE and human Pex5 (for further details see the interpretation section of Supplementary Fig. S1). These findings prompted us to experimentally test whether non-canonical motifs such as SYM can mediate peroxisomal import with detectable efficiency *in vivo*.

To investigate the role of the SYM motif in peroxisomal import, we generated a strain expressing SYM-tagged Gfp (Gfp-SYM; see Methods) and a peroxisomally localized red fluorescent protein tagged with SKL (DsRed-SKL). Co-localization of the Gfp signal with a co-expressed DsRed-SKL verified the peroxisomal localization of Gfp-SYM (Fig. 1b). Experimental results were supported by binding energy calculations of PexE-cargo complexes performed using the HADDOCK server (Fig. 1c).

### Occurrence and distribution of SYM-proteins in Kingdom fungi

Since the peroxisomal localization of Gfp-SYM confirmed that SYM can function as a peroxisomal targeting signal in *A. nidulans*, we extended our investigation to assess the prevalence of the SYM motif across fungi. An *in silico* analysis of 2,267 fungal species (retrieved from the Joint Genome Institute (JGI) MycoCosm fungal genome database^28,29^ in 2022) revealed that 853 species (37.6% of the dataset), distributed across 26 classes from seven fungal phyla, encode proteins with a C-terminal SYM tripeptide. This search identified a total of 1,413 SYM-proteins across 1,010 genomes (see Supplementary Data sheet ‘Fig_S3_sym_heatmap_detailed’ for details).

**Fig. 2:**
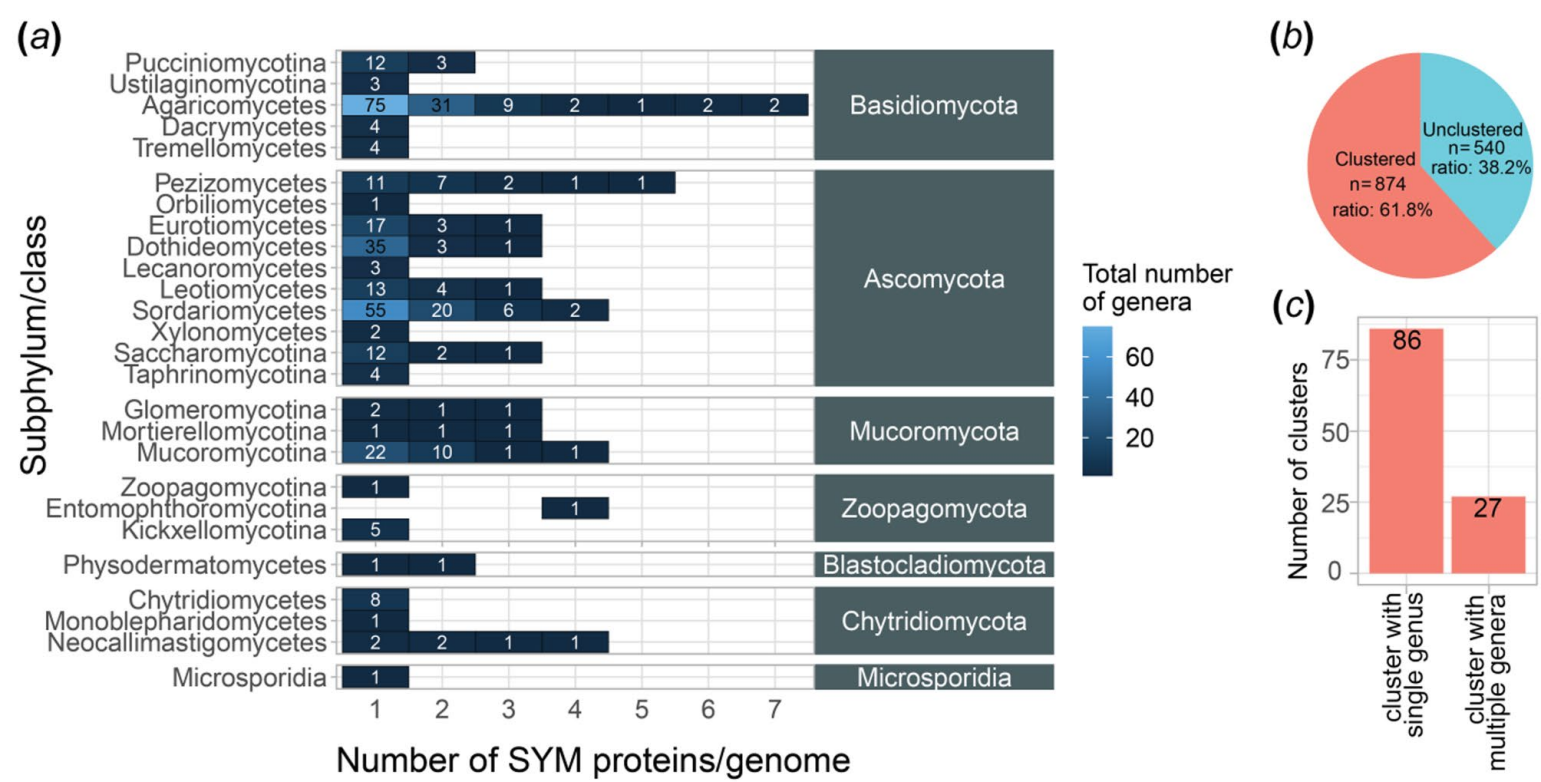
Distribution of SYM-proteins in fungi. (**a**) Number of SYM-proteins per genome across fungal kingdom. Columns represent the number of SYM-proteins per genome; rows represent various taxa; colors and numbers in the heat map denote the number of representing genera. (**b**) The pie chart shows the ratio of cluster-forming and unclustered SYM-proteins. (**c**) The barplot shows the number of clusters represented by a single or multiple genera. Number of clusters represented by a single genus exceeded the number of clusters with multiple genera.

Because certain fungal species and genera are overrepresented in the database, statistical analysis at the species level may result in biased interpretations. To mitigate this, we restricted our analyses to the genus level. We screened the proteomes of species from 354 genera, spanning seven fungal phyla: *Basidiomycota*, *Ascomycota*, *Mucoromycota*, *Zoopagomycota*, *Blastocladiomycota*, *Chytridiomycota* and *Microsporidia*. SYM-containing proteins were identified in 148, 208, 41, 7, 2, 15 and 1 genera from these respective phyla (Fig. 2a, Supplementary Fig. S3 and Supplementary Data sheet ‘Fig_2A_sym_heatmap’). Most genomes contain one or two SYM-proteins, while three to seven proteins were detected only in rare instances.

Among the 1,413 identified SYM-proteins, 540 were unique based on an orthology search, while the remaining 874 proteins clustered into 113 groups (Fig. 2b, Supplementary Data sheets ‘Fig_2B_clustered_seqs’ and ‘Fig_2B_unclustered_seqs’). These clusters consisted of SYM-proteins from different species within the same genus (Fig. 2c, Supplementary Data sheet ‘Fig_2C_cluster_types’), suggesting that SYM-proteins likely arise independently rather than through phylogenetic inheritance.

**Fig. 3:**
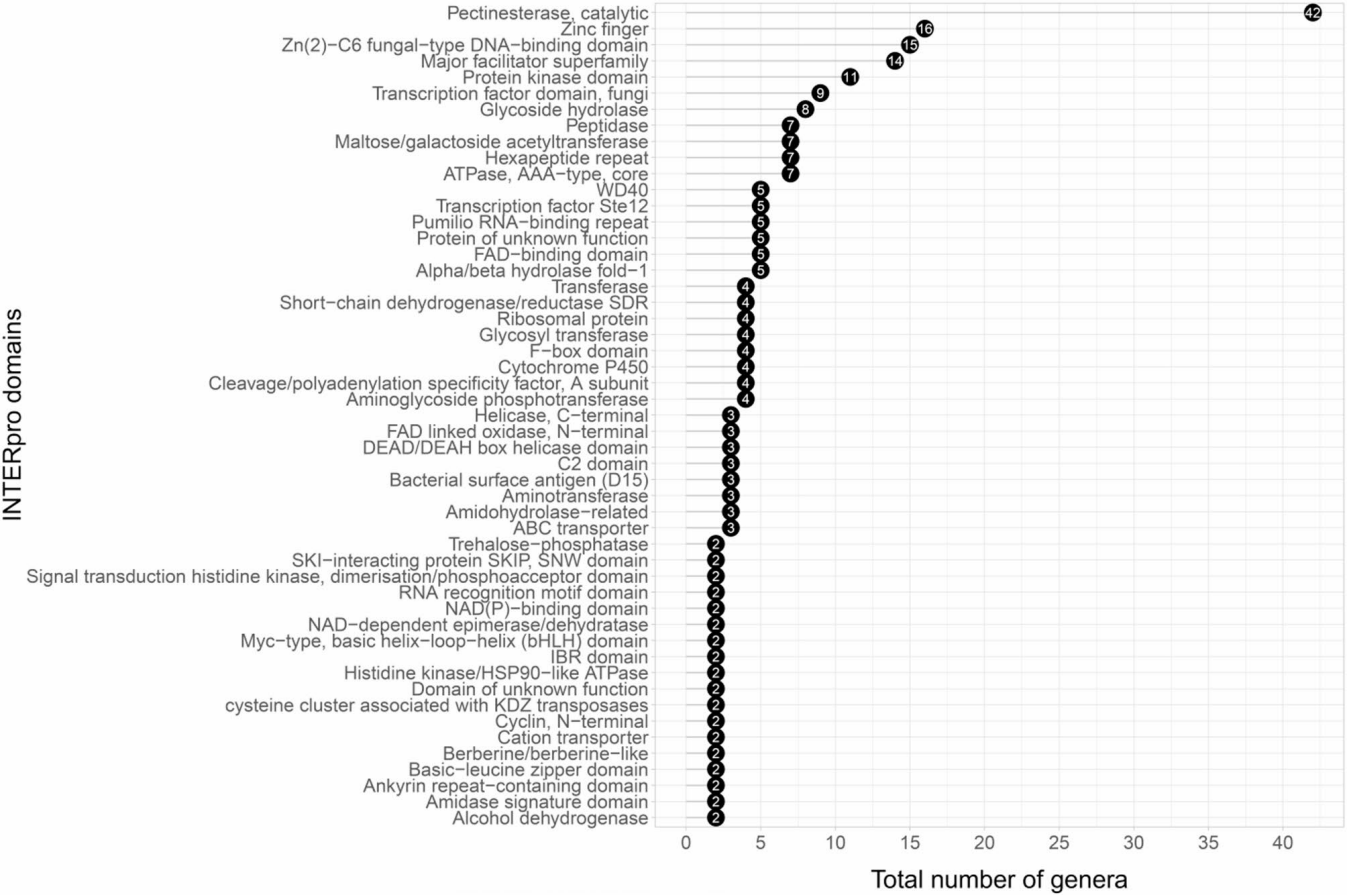
Interpro domain analysis of SYM-proteins. The figure shows 51 distinct protein domains with at least 2 representative genera harboring the corresponding functional domain on the investigated SYM-proteins. Altogether, a total of 181 distinct protein domains were identified across fungal SYM-proteins from 221 genera. Note that some genera contained multiple SYM-proteins with different domain architectures, which explains why the total number of genera represented in the figure exceeds the actual taxonomic count.

An InterPro domain analysis of the full set of SYM-proteins revealed a high degree of functional diversity (Fig. 3, Supplementary Data sheet ‘Fig_3_ipr’), further reinforcing the notion that, with few exceptions, SYM-proteins lack significant phylogenetic relationships. These findings strongly suggest that SYM-proteins may arise spontaneously within genomes. Pectinesterase activity was the most frequently identified function among SYM-proteins (Fig. 3, Supplementary Data sheet ‘Fig_3_ipr’).

**Fig. 4:**
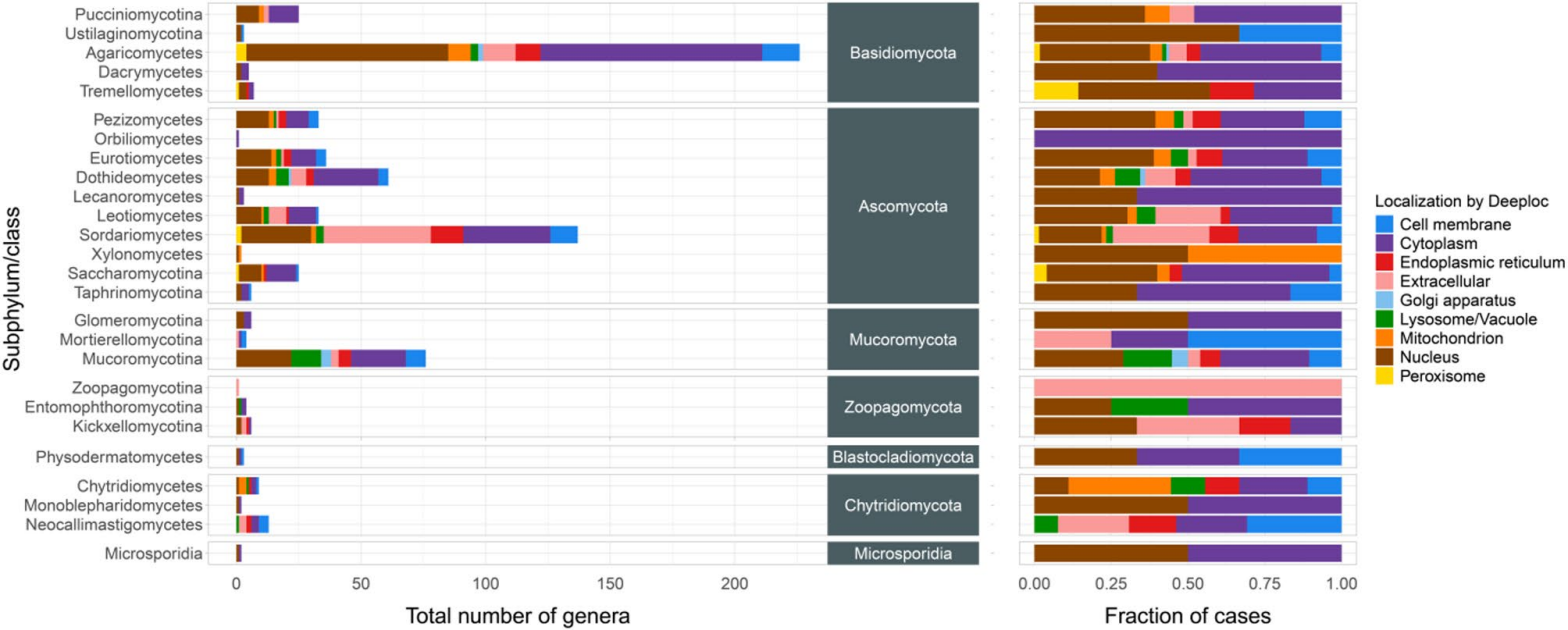
Occurrence of various intracellular locations in the main subhyla or classes of the seven fungal phyla. The left graph displays the number of genera within each subphylum or class of the seven fungal phyla that contain SYM-proteins with distinct localizations, as predicted by DeepLock 2.0. The right graph illustrates the relative intracellular distribution of SYM-proteins across the genera within the examined subphyla or classes.

Although, like other PTS-1 prediction tools (e.g., YLoc, WoLF PSORT II, PredPlantPTS1), DeepLock 2.0 failed to recognize the C-terminal SYM tripeptide as a PTS-1 signal in fungi, it is intriguing that the DeepLock 2.0 analysis predicted two-thirds of SYM-proteins to localize to one or more specific cellular compartments. These included the nucleus (48.5%), extracellular space (15.6%), cell membrane (6.58%), endoplasmic reticulum (5.02%), mitochondrion (3.89%), lysosome/vacuole (3.11%), and Golgi apparatus (0.5%) (Fig. 4, Supplementary Fig. S4, Supplementary Data sheet ‘Fig_4_deeploc’). Only 35.3% of SYM-proteins were predicted to be cytoplasmic (Fig. 4). Interestingly, 0.99% of SYM-proteins were predicted to be peroxisomal.

**Fig. 5:**
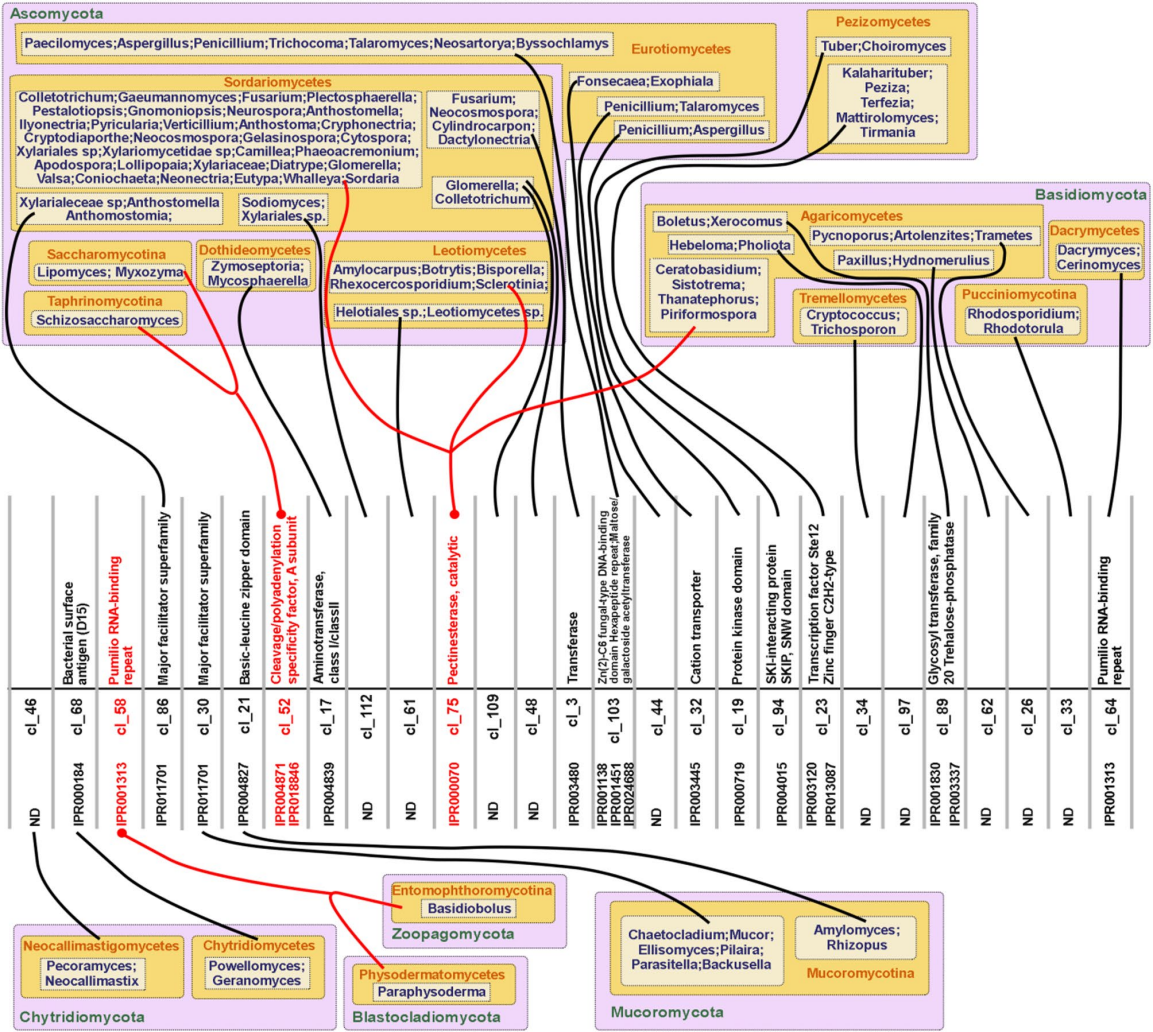
Clusters composed of SYM-proteins from more than one genus. The InterPro domain number and description are shown adjacent to the cluster list number. Representative genera for each cluster are displayed in boxes, organized by their respective class and phylum. Lines connect the representative genera groups to their corresponding cluster numbers. Clusters containing SYM-proteins from multiple subphyla or classes are highlighted with red lines and red descriptions.

In rare instances, clusters contained representatives from more than one genus (Fig. 5, Supplementary Fig. S5, Supplementary Data sheet ‘Fig_5_clusterplot’). Notably, only three SYM-proteins, which were functionally diverse, were found across more than one subphylum or class. This observation could indicate evolutionary conservation of the spontaneously acquired peroxisomal localization in these proteins (Fig. 5).

In cluster cl_58, the SYM-proteins were orthologs of a Pumilio RNA-binding repeat domain protein (IPR001313) found in representatives of two phyla, *Blastocladiomycota* and *Zoopagomycota*.

In cluster cl_75, the SYM-proteins were orthologs of a pectinesterase (IPR000070 domain) and were representatives of both *Basidiomycota* and *Ascomycota*. Interestingly, this pectinesterase SYM-protein was restricted to only one class within *Basidiomycota* (*Agaricomycetes*) and two classes within *Ascomycota* (*Sordariomycetes* and *Leotiomycetes*) (Fig. 5). However, the number of represented genera was relatively high, including 32 from *Sordariomycetes*, 5 from *Leotiomycetes* and 4 from *Agaricomycetes* (Fig. 5).

Orthologous SYM-proteins in cluster cl_52 (containing the IPR004871 domain) are distributed across two subphyla of the *Ascomycota* phylum, *Taphrinomycotina* and *Saccharomycotina*. These SYM-proteins represent three genera, including *Schizosaccharomyces* (Fig. 5). In *Schizosaccharomyces pombe*, the cl_52 protein is known as cleavage termination factor 1 (Ctf1p), a well-characterized protein that operates in the nucleus^30^. Ctf1p contains a cleavage and polyadenylation factor-interacting domain and functions as a Ran14- and Res2-interacting protein.

### SYM-proteins AN1402 and AN5316 in the model organism *Aspergillus nidulans*

The *A. nidulans* genome encodes two SYM-proteins. One of these, AN1402, is a 692 AA long putative transcription factor. AN1402 contains two nuclear localization signals (NLS) located at AA positions 101–111 and 259–280, a Zn2/Cys6 fungal-type DNA-binding domain (IPR001138) spanning AAs 267–302, and a Trimeric LpxA-like superfamily domain (IPR011004) covering AAs 491–682. This latter domain includes a Maltose/galactoside acetyltransferase domain (IPR024688) between AAs 492–549 and a Hexapeptide repeat domain (IPR001451) from AAs 642–674. DeepLock 2.0 predicted the nuclear localization of this protein with 94% probability.

AN5316 is a 194 AA long protein with no recognizable functional domains. DeepLock 2.0 predicted dual localization in the cytoplasm and nucleus with probabilities of 50.6% and 79.0%, respectively. Structural predictions using AlphaFold 3 (https://alphafoldserver.com/), I-Tasser^31^ and Phyre2^32^ identified wasp hyaluronidase (hydrolase found in wasp venom) (PDB codes: 2 atm) as the closest structural homolog of AN5316 with a Tm-score 0.642, RMSD of 3.63, 0.105% sequence identity in the structurally aligned region, and an alignment coverage of 0.871 (meaning that 87.1% of AN5316 residues aligned structurally).

To investigate the expression of AN1402 or AN5316, we used cDNA samples from our earlier studies, obtained from wild-type *A. nidulans* strains grown on glucose as a carbon source and various nitrogen sources (ammonium tartrate, acetamide, nitrate) or without a nitrogen source, at 37 °C for 8–10 hours^33,34^. Under these conditions, we found that neither AN1402 nor AN5316 were expressed. This finding aligns with results from other published transcriptome analyses performed under similar growth conditions^35,36^. Additionally, AN1402 and AN5316 were not detected in other transcriptome studies available through FungiDB (https://fungidb.org/)^37,38^. These studies used growth conditions different from ours, such as the use of cellulose as sole carbon source or RNA samples derived from resting conidiospores.

**Fig. 6:**
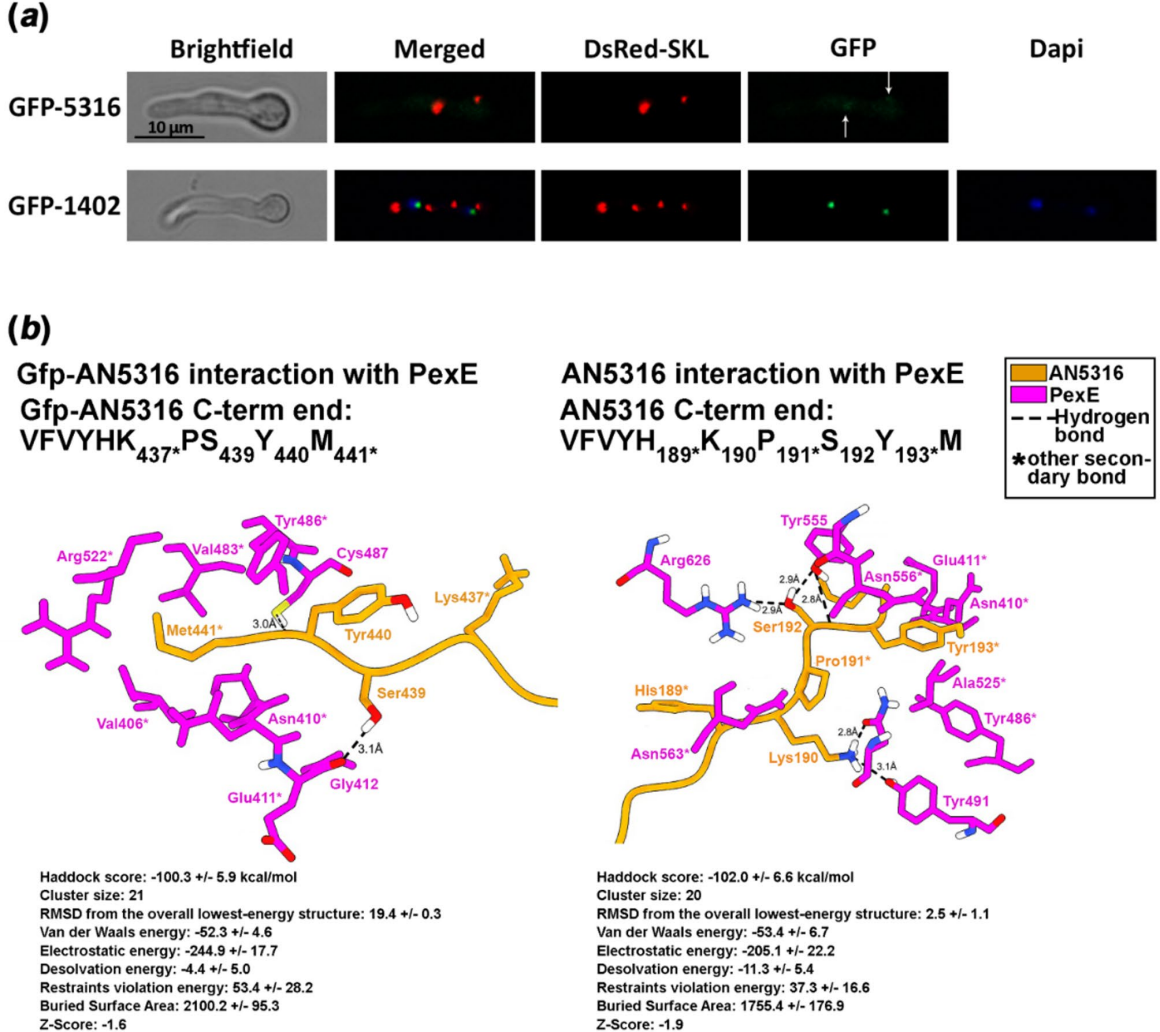
Subcellular localization analysis of AN5316 and AN1402. (**a**) Intracellular localization of Gfp-fused AN5316 (GFP-5316) overlaps with the co-expressed SKL-tagged red fluorescence protein (DsRed-SKL), indicating peroxisomal localization. Gfp-fused AN1402 proteins co-localize with DAPI stained nuclei. Fluorescent microscopy was performed using Zeiss 09, 15 and 49 filter sets for DsRed, Gfp and Dapi, respectively. Conidia were germinated for 6.5 h at 37 °C on coverslips submerged in MM followed by imaging. Scale bar: 10 μm. The content of the produced Gfp-5316 in strain HZS.1041 was determined by Western blot (Supplementary Fig. S2). (**b**) Interaction of *A. nidulans* PexE (magenta) with Gfp-AN5316 (orange) (left) and AN5316 (orange) (right). Hydrogen bonds are indicated with dashed lines, and distances between interacting atoms are given in Ångströms. Asterisks denote van der Waals or other secondary interactions. For each PexE-cargo complex, the sequence of the C-terminal decapeptide is shown, with the interacting residues in the cargo indicated by subscripted positions. Binding energy (in kcal/mol) was calculated using the HADDOCK server based on the full-length sequences of PexE and the respective cargo proteins. Detailed HADDOCK results are provided below the figures. More negative kcal/mol values indicate stronger interactions.

Given the lack of information on the timing and environmental conditions required for the physiological expression of AN1402 and AN5316, we investigated the subcellular localization of these gene products using N-terminal Gfp fusions. To bypass the challenge of low or conditional expression, we used the constitutive promoter of *gpdA* (P*gpdA*) to drive the expression of the Gfp fusion constructs, rather than relying on the native promoters of the target genes. These fusion constructs were expressed in a strain with peroxisomes labeled by DsRed-SKL, a red fluorescent protein tagged with the SKL PTS-1 signal. The Gfp-AN1402 fusion protein exhibited exclusive nuclear localization, whereas the Gfp-AN5316 fusion protein displayed faint cytoplasmic and peroxisomal localization (Fig. 6a). To verify the production and intracellular abundance of the Gfp fusion protein, we performed Western blot analysis on whole-cell extracts of the Gfp-AN5316 expressing strain (Supplementary Fig. S2). Given that a substantial portion of the fusion protein is supposedly distributed throughout the relatively large cytoplasmic volume, the weak cytoplasmic fluorescence signal is plausible. This interpretation is supported by other studies, in which a defined amount of fusion protein produces a strong signal in a confined compartment (e.g. nucleus), while the same amount yields barely detectable fluorescence when dispersed in the cytoplasm^39^. To support the Gfp-fusion experiments (Fig. 6a), binding energy calculations of PexE-Gfp-AN5316 and PexE-AN5316 complexes were performed using the HADDOCK server (Fig. 6b). The generated data indicate that Gfp fusion weakened the cargo protein interaction with PexE. While AN5316 could establish five Hydrogen bonds through Ser (at position − 3) and Lys (at position − 5), the Gfp-fused AN5316 could establish two Hydrogen bonds through Ser (at position − 3) and Tyr (at position − 2) (detailed HADDOCK results are included) (Fig. 6b).

The weak peroxisomal fluorescence of Gfp-AN5316 is consistent with previous observations of plant proteins carrying non-canonical PTS-1 signals, which often exhibit weak or undetectable peroxisomal localization, with the majority of the protein localized elsewhere in the cell^12,15^. In the case of Gfp-AN1402, the nuclear localization signals of AN1402 directed the fusion protein to the nucleus, which is likely the primary location for this zinc finger domain-containing protein.

Although the Gfp-AN1402 fusion construct was expressed from a strong constitutive promoter, leading to potentially non-physiological levels of the protein, we hypothesized that, under specific environmental stimuli, the native AN1402 protein might shift from nuclear to cytoplasmic localization. To investigate this, we pre-cultured the Gfp-AN1402-expressing strain on coverslips submerged in glucose-nitrate minimal medium for 6 h at 37 °C. Following this, the medium was replaced with various media containing different carbon sources (1% (m/V) lactose, 2% (V/V) ethanol), nitrogen sources (5 mM ammonium tartrate, 1 mM acetamide, or no nitrogen source), or glucose-nitrate medium supplemented with stress-inducing agents. The stressors included oxidative stress agents (0.05, 0.01 and 0.25 mM menadione, 0.4, 1.6 and 2 mM diamide, 40 mM H₂O₂), cell wall stressor (1% SDS), a heavy metal stress agent (50 µM CdSO₄), and osmotic stress agents (2 M NaCl, 2 M sorbitol). We also tested heat stress conditions at 42 °C. After further incubation for two hours at 37 °C, the Gfp-AN1402 fusion protein showed only nuclear localization under all tested conditions. Consequently, the role and mechanism of action of this transcription factor remain unknown.

## Discussion

Extensive proteomic studies on plant peroxisomes have revealed numerous non-canonical PTS-1 signals that interact weakly with the Pex5 receptor, resulting in slow peroxisomal import of the associated proteins^15^. The concept of “modified or inefficient peroxisomal targeting signals” as proposed by Ast et al. suggests that such signals may enable dual localization of proteins that are otherwise targeted to specific compartments (such as the cytoplasm, mitochondrium, nucleus, vacuolum) to both their primary location and peroxisomes^40^ and references therein). One such example is the SYM motif, originally described as a non-canonical PTS-1 signal, which was shown to form weak interactions with Pex5 in plants, resulting in slow import and partial peroxisomal localization^12,15^. Although currently available PTS-1 prediction tools fail to recognize fungal SYM-proteins as peroxisomal, we demonstrated using SYM-tagged Gfp that this non-canonical motif functions as a valid peroxisomal targeting signal in *A. nidulans*. Moreover, we showed that the endogenous SYM-protein AN5316 exhibits dual localization, predominantly cytoplasmic, with weak peroxisomal targeting. These findings align with earlier work of Reumann et al.^15^ and theoretical framework of Ast et al.^40^. Notably, residues upstream to the C-terminal tripeptide are critical in determining the strength of interaction between non-canonical PTS-1 signal and the TPR domain of Pex5. Kragler et al.^41^ and Elgersma^42^ et al. provided key examples: a non-canonical SKF-tagged luciferase (with Phe at position − 1 as non-canonical AA) failed to localize to peroxisomes in yeast, whereas SKF-tagged yeast MDH3 (encoding malate dehydrogenase 3) localized efficiently to peroxisomes. Similarly, yeast Idp3 (encoding isocitrate dehydrogenase 3), which carries the non-canonical PTS-1 signal CKL (Cys at position − 3 as non-canonical AA), localized exclusively to the peroxisome, whereas CKL-tagged Idp2 (encoding isocitrate dehydrogenase 2) showed dual, cytoplasmic and weak peroxisomal localization^25^. Idp2 was originally described as cytoplasmic enzyme, however, this enzime exhibits a C-terminal AAL tripeptide, which constitutes a non-canonical PTS-1 signal (Ala at -2 position as non-canonical AA). Based on our current understanding of non-canonical PTS-1 signals, it cannot be excluded that the Gfp-Idp2 fusion is imported into the peroxisomes in amounts below the detection threshold of fluorescent microscope, suggesting that Idp2 may have a physiologically dual localization, both cytoplasmic and peroxisomal.

In a related context, Alves de Castro et al. recently linked gliotoxin self-defense in *Aspergillus fumigatus* to a peroxisomal function. Although two candidate proteins, GliT and GmtA, were implicated in this self-defense mechanism, Gfp phusion experiments did not support their peroxisomal localization^43^. Moreover, no PTS-1 signals were identified in these proteins using *in silico* predictor tools^43^. Notably, the C-terminal QEL tripeptide of GliT conforms to a non-canonical PTS-1 signal (Gln and Glu at positions − 2 and − 3 are non-canonical AAs, respectively). However, the authors, seemingly unaware of Reumann’s PTS-1 criteria, did not test whether truncation of the QEL sequence affects gliotoxin sensitivity, an experiment that could clarify a potential peroxisomal role for GliT.

We investigated the localization of a second SYM-protein, a transcription factor coding AN1402, in *A. nidulans*. The constitutively expressed Gfp-AN1402 showed nuclear localization under various environmental conditions. However, the absence of observed peroxisomal localization for this protein does not exclude the possibility that AN1402 may translocate to peroxisomes under specific conditions, including such that were not involved in our investigations. We propose that such translocation may regulate protein levels during their initial synthesis or when shuttling between the nucleus and cytoplasm. Although investigations of nuclear/peroxisomal dual localization of transcription factors are relatively rare, notable examples exists in the literature. For instance, the peroxisomal sequestration of HIF-1, a Hypoxia-Inducible Factor, in hepatocytes^44^ suggests that peroxisomal internalization may regulate transcription factor activity. This observation supports our hypothesis that the peroxisomal localization of AN1402 could serve a functional role, potentially acting as a regulatory mechanism under specific physiological or stress conditions.

Whether the weak peroxisomal localization of AN5316 or the hypothesized peroxisomal translocation of AN1402 under specific physiological conditions has functional importance remains unclear. To date, these proteins lack assigned functions, and little is known about the timing and regulation of their expression. Further investigations are needed to understand the context in which these proteins localize to peroxisomes and to determine whether this localization has functionally importance, especially considering the limited knowledge about their roles and expression dynamics.

We also considered the evolutionary origins of non-canonical PTS-1 signals through *in silico* analysis of SYM-proteins. These proteins are rare across translated fungal genomes, with typically only one or two SYM-proteins encoded per genome. Functional and subcellular localization analyses of 1,413 SYM-proteins revealed striking diversity, particularly among phylogenetically unrelated species. This diversity likely reflects the spontaneous emergence of C-terminal SYM tripeptides via point mutations. While previous studies have proposed only theoretical models for the spontaneous emergence of PTS-1 motifs, without any empirical evidence^15^ our findings provide indirect empirical support for the hypothesis that PTS-1 signals can arise spontaneously through mutational processes.

In fungi, two-thirds of SYM-proteins localize primarily to the nucleus and cytoplasm, but they are also detected in other compartments such as the Golgi apparatus, endoplasmic reticulum, cell membrane, mitochondria, or lysosomes. If the SYM motif confers additional peroxisomal localization, these proteins should be regarded as dually localized, exhibiting weak interactions with Pex5 receptor. This interpretation aligns with the model proposed by Ast et al., which posits that weak Pex5 binding facilitates dual localization^40^. We propose that such dually localized SYM-proteins (and other non-canonical PTS-1-carrying proteins) may not immediately acquire functional roles through partial peroxisomal localization. Instead, they might serve as latent reserves within peroxisomes, available to support novel or repurposed biological processes during future evolutionary adaptation. This latent potential may present a flexible evolutionary toolkit, facilitating innovation and adaptation to environmental challenges.

Bioinformatics analysis of SYM-proteins revealed a high frequency of pectinesterase domains, suggesting a potential importance of peroxisomal localization in pectin metabolism. We hypothesize that the SYM-pectinesterase orthologs across *Ascomycota* (*Sordariomycetes* and *Leotiomycetes*) and *Basidiomycota* (*Agaricomycetes*) arose through an evolutionary processes. During this process, a spontaneous point mutation likely introduced a C-terminal SYM motif into an ancestral pectinesterase, leading to partial peroxisomal localization. This change may have conferred an evolutionary advantage, either immediately or gradually, by enabling the organism to explore novel functional contexts for the enzyme, ultimately contributing to its evolutionary success.

Fungal pectinesterases are pivotal in plant pathogenesis, catalyzing the de-esterification of pectin, a major component of plant cell walls^45–48^. Phylogenetic analyses reveal that genes encoding these enzymes underwent multiple duplication events during evolution^49^. The resulting paralogs likely reduced the functional constraints on ancestral pectinesterases, making the acquisition of a SYM PTS-1 signal an evolutionary low-risk event. This redundancy allowed the newly evolved SYM-pectinesterase to localize partially to peroxisomes without impairing essential functions of the paralogs, conferring an adaptive advantage with minimal immediate cost.

We hypothesize that partial peroxisomal localization of SYM-pectinesterases could play a key role in plant pathogenesis, akin to the dual localization of Gapdh (glyceraldehyde-3-phosphate dehydrogenase) and Pgk1 (3-phosphoglycerate kinase) in *Ustilago maydis*^7^. The compartmentalization of Gapdh and Pgk1 in *U. maydis* underscores the potential evolutionary significance of non-canonical PTS-1 signals, including the SYM motif. Gapdh and Pgk1, initially thought to be exclusively cytoplasmic, were later discovered to harbor cryptic PTS-1 signals that are undetectable though gene sequence analysis alone. These functional PTS-1 signals arise though mechanisms such as alternative splicing (Gapdh) or ribosomal read-through (Pgk1), enabling a small subset of the proteins to localize to peroxisomes. This partial localization is challenging to detect using Gfp-tagged fluorescence microscopy^7^. Similarly, SYM-pectinesterases, characterized by having weak, non-canonical SYM PTS-1 signals, may have evolved unbalanced dual localization, with only a fraction of the protein pool entering peroxisomes. In *U. maydis*, the partial peroxisomal localization of Gapdh and Pgk1 is critical for virulence, as disrupting their PTS-1 targeting significantly reduces virulence^7^. By analogy, the weak peroxisomal localization of SYM-pectinesterases might also be crucial for plant pathogenesis, highlighting their functional importance during infection.

The proposed role of SYM-pectinesterases in plant pathogenesis may explain the conservation of the SYM signal in pectinesterase orthologs across *Ascomycetes* and *Basidiomycetes*. This evolutionary conservation likely reflects selective pressure to maintain the partial peroxisomal localization of pectinesterases, which could be crucial for virulence and fungal interactions with the plant hosts. If SYM-pectinesterases indeed play a role in pathogenesis, it is plausible that the SYM motif is not the sole non-canonical PTS-1 signal enabling partial peroxisomal localization of pectinesterases. We explored this possibility by conducting a systematic *in silico* analysis of available fungal pectinesterase sequences from the JGI database to identify non-canonical PTS-1 signals - besides known canonical ones - that may have emerged during fungal evolution (Table 1, Supplementary Data sheet ‘‘Table_1’’ ). This analysis allowed us to determine if other non-canonical PTS-1 signals, beyond the SYM motif, have independently evolved in fungal pectinesterases, potentially contributing to their compartmentalization and functional roles in pathogenesis.

**Table 1:** Occurrence of canonical and non-canonical PTS-1 tripeptides at the C-terminus of fungal pectinesterases according to the reumann’s plant formula [A/S/(Q/I/K/L/T/G/V/F/C/P)] [K/R/(A/D/Y/C/Q/P/F/T/E/G/H/M/L/N/S)] [I/M/L/(F/Y/V)] proposed by Reumann et al.^14^.

Formula*	Number of pectin-esterases	Detected tripeptide
**C**_**− 3**_ **C**_**− 2**_ **C**_**− 1**_	1	-**ARI**
**C**_**− 3**_ **C**_**− 2**_ NC_− 1_	27	-**AK**V, -**AR**(F/Y), -**SK**Y, -**SR**(V/Y)
**C**_**− 3**_ NC_− 2_ **C**_**− 1**_	447	-**A**(G/N/Y/A/D/F/C/Q)**L**, -**A**(L/M/Y/T/F)**M**, -**A**Y**I**, -**S**(G/F/Y/P/H/S)**L**, -**S**(Y/F)**M**, -**S**(S/Y)**I**
**C**_**− 3**_ NC_− 2_ NC_− 1_	245	-**S**(A/H/P/L/N/Y/E/S/D/T/F/G)V, -**S**(S/Y/T)F, -**S**(S/G/T/L)Y, -**A**(A/L/D/Y/E/S/T/N)Y, -**A**(D/Y/E)V, -**A**(P/G/S)F
NC_− 3_ **C**_**− 2**_ **C**_**− 1**_	10	-(I/G/L/F)**RI**, -(I/G)**KI**, -(C/G)**RL**, -K**RM**, -I**KM**
NC_− 3_ **C**_**− 2**_ NC_− 1_	18	-G**K**(F/V), -(P/T)**R**V, -(T/I)**K**Y, -L**K**V
NC_− 3_ NC_− 2_ **C**_**− 1**_	284	-I(S/E/G)**L**, -Q(P/L/D/C)**L**, -V(S/N/F/H/E/P/G)**L**, -G(P/G/D/Y)**L**, -F(S/G/L)**L**, -L(S/N/F/P/G/A/T/Y)**L**, -K(F/H/L/A/Y)**L**, -T(S/Y/F)**L**, -P(H/E/Y/G)**L**, -I(L/C)**I**, -C(L/Y)**I**, -Q(H/A/T)**I**, -VG**I**, -G(S/N/G/L/A/T/Y)**I**, -F(S/T/Y)**I**, -L(S/P/G/L)**I**, -K(S/F/C/Y)**I**, -TY**I**, -P(S/T)**I**, -IN**M**, -CS**M**, -Q(S/F)**M**, -V(L/C)**M**, -G(D/T)**M**, -FM**M**, -LT**M**, -KY**M**, -T(L/Y)**M**, -PD**M**
NC_− 3_ NC_− 2_ NC_− 1_	308	-G(Q/P/E/F/N/T/A)V, -T(T/S/Y)V, -F(T/S/D)V, -KNV, -V(S/L/C)V, -L(G/T/S/E)V, -P(F/D/S/E)V, -Q(S/C)V, -G(T/S/G/D/L)Y, -T(L/E/S/T/A)Y, -F(S/L/P/T/H)Y, -K(F/S/T/A/Y)Y, -I(T/S)Y, -L(L/S/F/P)Y, -P(L/S)Y, -Q(S/F)Y, -CNY, -G(Y/G/H/S)F, -T(S/Y/D)F, -F(T/D/L)F, -P(S/E)F, -IDF, -V(G/L/P)F, -L(A/T/S/D)F, -QDF-

Bold letters denote canonical amino acids. * C: canonical amino acid; NC: non-canonical amino acid; subscript denotes the position of the amino acid in the C terminal PTS-1 signal tripeptide.

Remarkably, among the 5,250 fungal pectinesterases analyzed, 1,341 (25.5%) contained either verified or proposed canonical or non-canonical PTS-1 signals. Most of these signals comprised non-canonical tripeptides, including motifs with two canonical and one non-canonical amino acid (*n* = 447), one canonical and two non-canonical amino acids (*n* = 529), or three non-canonical amino acids (*n* = 308) (Table 1).

The spontaneous acquisition of partial peroxisomal localization via point mutations generating non-canonical PTS-1 motifs serves as compelling evidence of convergent evolution in the compartmentalization of pectinesterases. This pattern indicates that evolutionary pressure to localize these proteins partially to peroxisomes (potentially for roles in pathogenesis or other biological processes) has independently driven the emergence of diverse non-canonical PTS-1 signals across fungal lineages.

Convergent evolution toward partial peroxisomal compartmentalization, as seen in SYM-pectinesterases, has also been documented for fungal orthologs of Gapdh and Pgk1 from *U. maydis*. In this species, Gapdh acquires its PTS-1 signal through alternative splicing, whereas Pgk1 gains peroxisomal access via ribosomal read-through. Intriguingly, in other fungi, such as *A. nidulans*, the mechanisms are reversed, Gapdh utilizes ribosomal read-through, while Pgk1 employs alternative splicing^7^. Further evidence of convergent evolution in peroxisomal targeting comes from *Phycomyces blakesleeanus* (*Zygomycota*), where two of its three Gapdh paralog genes acquired non-canonical PTS-1 signals, -GAL and -GNL, with the latter confirmed as functional^7^.

In summary, this study supports the hypothesis that non-canonical PTS-1 signals can arise spontaneously through evolutionary processes, while highlighting the extensive diversity of SYM-proteins. These findings emphasize the broader importance of dual or partial peroxisomal localizations across various proteins. Such partial compartmentalization likely enhances functional plasticity, offering an evolutionary advantage by enabling proteins to operate in multiple cellular contexts and adapt to diverse environmental pressures over time.

## Methods

### Strains and media

Standard *Aspergillus* genetic markers, complete medium (CM) and minimal medium (MM) are described at the following URL: http://www.fgsc.net/Aspergillus/gene_list/. Strains were maintained on CM, while MM supplemented with required vitamins (www.fgsc.net) was used for transformation, strain purification and culturing for microscopy. Strain *pantoB100 biA1 pabaA1 veA1* carrying 1 copy of *pDsRed-SKL-argB*^21,27^ plasmid (developed in this work from NA1322^21^ by genetic crossing) was used as recipient strain for transformation of non-tagged *gfp*-, SYM- and SRL-tagged *gfp*-expression vectors. This strain expresses canonic PTS-1 signal (SKL)-tagged red fluorescence protein (DsRed) under the control of the constitutive *gpdA* promoter (P_*gpdA*_), enabling red fluorescence emission for the peroxisomes. Strains HZS.948, HZS.949 and HZS.952 were developed by transforming the NA1322 originated recipient strain with the below described *gfp*, *gfp*-SYM and *gfp*-SRL expressing plasmids pAN-HZS-1, pAN-HZS-23 and pAN-HZS-22, respectively. Strain HZS.573 (plasmid of *pDsRed-SKL-argB* integrated in one copy, *pantoB100 pabaA1 biA1 veA1*) was used to generate Gfp-AN1402 and Gfp-AN5316 fusion protein-expressing strains (HZS.1022 and HZS.1041, respectively), by transformation with the below described pAN-HZS-30 and pAN-HZS-29 expression plasmids.

### Construction of *gfp*-tagged (C-terminal tagged) expression vectors

The SYM- and SRL-tagged Gfp expressing constructs were generated by cloning C-terminal SYM- and SRL-tagged (encoded by AGT TAT ATG and TCG CGG TTA sequences) *gfp* PCR product (amplified by “gfp NcoI start frw” (5’-ttttttttccatggtgagcaagggcgaggagc-3’) - “gfp SYM BamHI rev” (5’-ttttttttggatcctta*catataact*cttgtacagctcgtccatgccg-3’) and “gfp NcoI start frw” - “gfp SRL BamHI rev (5’-ttttttttggatcctta*taaccgcga*cttgtacagctcgtccatgccg-3’) primer pairs) into NcoI-BamHI digested pAN-HZS-1 *Escherichia coli*/*A. nidulans* shuttle expression vector^50^. The resulting SYM- and SRL-tagged Gfp-expressing vector (pAN-HZS.23 and pAN-HZS.22, respectively) were constructed so that tagged *gfp* was driven by the constitutive *gpdA* promoter (P_*gpdA*_) and terminated by the *trpC* terminator (T_*trpC*_). These vectors also contain the wild type *pantoB* gene from *A. nidulans* for selection purposes following *A. nidulans* transformation. For vector construction, *E. coli* JM109^51^ was used for transformation according to Hanahan^52^. Plasmid extraction and other DNA manipulations were performed following standard protocols as described by Sambrook^53^.

### Construction of N-terminal tagged *gfp*-AN1402 and *gfp*-AN5316 expressing vectors

To obtain *gfp*-AN1402 and *gfp*-AN5316 fusions, PCR products of *gfp* and the candidate genes were fused by using the Double-Joint PCR (DJ-PCR) method^54^. Coding sequence of *gfp* (722 bp long) was amplified from a pAN-HZS-1 vector template^50^ using the “1pGpd int frw” (5’-cagtatattcatcttcccatccaagaac-3’) and “10GFP linker rev” (5’-*atcaagatcgactgtatcaataagcttgt*acagctcgtccatgccgtg-3’) primers. Italicized letters at the 5’ end on the latter primer confer to a 24 bp long linker sequence encoding the linker amino acids (LIDTVDLD), while the 3’ end is specific to the 3’ end of *gfp* excluding the stop codon. The 2458 bp and 1016 bp coding sequence of AN1402 and AN5316, respectively, were amplified from a wild-type strain (HZS.145) using the primer pairs “linker kim AN1402 frw” (5’-acaag*cttattgatacagtcgatcttgatatg*accgcaatcgcgaccgcatc-3’) - “AN1402 down rev” (5’-gtaactccgcagcaactagcaatcg-3’) and “linker kim AN5316 frw” (5’-acaag*cttattgatacagtcgatcttgatatg*tccagtccgaggcccccaaaac-3’) - “AN5316 down rev” (5’-cgacggtaggcgactggttaatg − 3’), respectively. 3’ ends of the forward primers were specific to the 5’ terminus of AN1402 or AN5316 (including the start codon; underlined letters), while the 5’ ends included the 8 amino acid linker sequence (italicized in the sequence). The amplified *gfp* were then combined with the coding sequences of AN1402 and AN5316 into a single fusion molecule using the DJ-PCR method. Nested forward and reverse primers were used for the fusion: “5GFP NcoI start frw” (5’- ttttttttccatggtgagcaagggcgaggagc − 3’) paired with “AN1402 NotI rev” (5’-ttttttttgcggccgctcacatatagcttggttttagtccctg-3’) and “AN5316 BamHI rev”(5’-ttttttttggatccctacatgtacgacggcttgtggc-3’), respectively. These primers, as their names indicate, included either NcoI, NotI or BamHI restriction sites at their 5’ ends. The resulting 2942 bp and 1787 bp long *gfp*-AN1402 and *gfp*-AN5316 fusion PCR products were cloned into an NcoI-NotI and NcoI-BamHI digested pAN-HZS-1 expression vector^50^. The resulting vectors, named pAN-HZS-30 and pAN-HZS-29, expressed the *gfp*-AN1402 and *gfp*-AN5316 fusion proteins, respectively, under the control of the constitutive P_*gpdA*_ promoter. These vectors also contained the *pantoB*^+^ gene as selection marker gene for transformation.

The plasmids pAN-HZS-30 and pAN-HZS-29 were transformed into a peroxisome labeled strain (HZS.573) expressing DsRed-SKL^21,27^. Pantothenic acid prototroph transformants were selected and successful integration of the *gfp*-fusion constructs was verified by PCR using the above described *gfp* specific primer pair “1pGpd int frw” and “10GFP linker rev”. Transformants containing two copies of the *gfp*-AN1402 and *gfp*-AN5316 integrations (HZS.1022 and HZS.1041, respectively) were selected for fluorescence microscopy analysis to observe the intracellular localization of the fusion proteins.

### PCR

The copy number of *gfp*-AN1402 and *gfp*-AN5316 integrations were determined on total DNA extract of the transformed strains^55^ by qPCR using *gfp* and gamma-actin specific primer pairs (“GFP ReTi frw” 5’-atcttcttcaaggacgacgg-3’ and “GFP ReTi rev” 5’-ttgaagtcgatgcccttcag-3’; “actin ReTi frw2” 5’-accatgtaccctggtatctc-3’ and “actin ReTi rev2” 5’-ggaggagcaatgatcttgac-3’, respectively).

Gene expression of AN1402 and AN5316 was studied using reverse transcription-qPCR (RT-qPCR) on various cDNA samples derived from our earlier studies (for details see the main text). The primer pair for the reference gene gamma actin was “actin ReTi frw” 5’- ggtatcatgatcggtatggg − 3’ and “actin ReTi rev” 5’- tatctgagtgtgaggatacca − 3’. Primer pairs for AN1402 and AN5316 were “AN1401 ReTi frw” 5’- gaccacagtatcaaagaccattcc − 3’, “AN1401 ReTi rev” 5’- agattaagcacaaactcctgatcc − 3’ and “AN5316 ReTi frw” 5’- gcgattcacattcacaccag − 3’ and “AN5316 ReTi rev” 5’- atcttcataaactctgccagcc − 3’. Transcript levels of of AN1402 and AN5316 were estimated using the relative standard curve method^56^with gene expression normalized to the reference gene gamma-actin.

### Transformation of *A. nidulans*

Protoplasts of *A. nidulans* recipient strain were prepared from mycelia grown on cellophane^57,58^ using a 4% solution of Glucanex (Novozymes, Switzerland) in 0.7 M KCl. Transformations were performed on 5 × 10^6^ and 5 × 10^7^ protoplasts using 1 µg of plasmid vectors, following the procedure described by Antal et al.^59^.

### Fluorescent microscopy

10^4^ conidiospores were inoculated onto the surface of coverslips submerged in supplemented MM and incubated at 37 °C for 5–6 h. Primary hyphae were then examined by fluorescence microscopy using Zeiss Axiolab A microscope. Zeiss filter sets 09, 15 and 49 were used to visualize DsRed, GFP and Dapi fluorescence, respectively.

### Protein extraction and Western-blot

Approximately 500 mg of mycelia of 20-h-old liquid MM cultures were grounded in liquid nitrogen and protein was extracted by using native extraction buffer (50 mM Tris pH 7.5, 0.1 M NaCl, 1.5 mM PMSF). Protein extracts were clarified by centrifugation (12 000 g, 5 min, 4 °C). Protein content of the supernatants was determined according to Bradford^60^. 20 µg of protein samples and 10 µl PageRuler Plus Protein Molecular Weight Marker (Thermo) were separated on 10% SDS-PAGE prepared according to Laemmli^61^. Proteins were transferred to PVDF (polyvinyldene fluoride) membrane (Millipore, Immobilion-P) using iBlot dry blotting system (Invitrogen, USA). GFP was detected using a GFP-specific antiserum (Takara, Living Colors A.v. Monoclonal Antibody (JL-8)) at a dilution of 1:1000. Secondary antibodies coupled to horse-radish peroxidase (Invitrogen, Goat anti-Mouse IgG (H + L) Secondary Antibody) were used at a dilution of 1:10 000.

### *In Silico* work

Subcellular localization of proteins containing the terminal SYM motif was predicted using DeepLoc version 2.0^62^. Clustering of proteins based on similarity was performed using MMseqs2 software release 14-7e284^63^. The analysis was conducted with 70% bidirectional overlap and a minimum similarity of 50% as criteria. Functional analysis of proteins was performed using interproscan version 5.60-92.0^64^. The search was restricted to using only the Pfam and PANTHER databases.

Protein structures were generated by using Alphafold server (https://alphafoldserver.com/). Structural homolog search was performed by using Alphafold-, I-Tasser^31^-, and Phyre2 ^32^ servers. Comparative structural analysis was conducted by using ChimeraX 1.3. Simulation of the interaction between Pex5 TPR domain and various cargo proteins were performed using the HADDOCK 2.4 server (https://rascar.science.uu.nl/haddock2.4)^65,66^ with default parameters, except that the active residues in the TPR domain cavity were set to “not buried” to avoid hindrance of the cargo proteins’ terminal signal motifs.

## Supplementary Information

Below is the link to the electronic supplementary material.

Supplementary Material 1

Supplementary Material 2

## Data Availability

Data is provided within the manuscript or supplementary information files.

## References

[1] De Duve, C. & Baudhuin, P. Peroxisomes (microbodies and related particles). *Physiol. Rev.* **46**, 323–357. 10.1152/physrev.1966.46.2.323 (1966). 5325972 10.1152/physrev.1966.46.2.323

[2] Bittner, E., Stehlik, T. & Freitag, J. Sharing the wealth: the versatility of proteins targeted to peroxisomes and other organelles. *Front. Cell. Dev. Biol.* **10**, 934331. 10.3389/fcell.2022.934331 (2022). 36225313 10.3389/fcell.2022.934331 PMC9549241

[3] Girzalsky, W., Saffian, D. & Erdmann, R. Peroxisomal protein translocation. *Biochim. Biophys. Acta*. **1803**, 724–731. 10.1016/j.bbamcr.2010.01.002 (2010). 20079383 10.1016/j.bbamcr.2010.01.002

[4] Emanuelsson, O., Elofsson, A., von Heijne, G. & Cristobal, S. *Silico* prediction of the peroxisomal proteome in fungi, plants and animals. *J. Mol. Biol.* **330**, 443–456. 10.1016/s0022-2836(03)00553-9 (2003). 12823981 10.1016/s0022-2836(03)00553-9

[5] Petriv, O. I., Tang, L., Titorenko, V. I. & Rachubinski, R. A. A new definition for the consensus sequence of the peroxisome targeting signal type 2. *J. Mol. Biol.* **341**, 119–134. 10.1016/j.jmb.2004.05.064 (2004). 15312767 10.1016/j.jmb.2004.05.064

[6] Kempinski, B. et al. The peroxisomal targeting signal 3 (PTS3) of the budding yeast Acyl-CoA oxidase is a signal patch. *Front. Cell. Dev. Biol.* **8**, 198. 10.3389/fcell.2020.00198 (2020). 32292783 10.3389/fcell.2020.00198 PMC7135854

[7] Freitag, J., Ast, J. & Bolker, M. Cryptic peroxisomal targeting via alternative splicing and stop codon read-through in fungi. *Nature* **485**, 522–525. 10.1038/nature11051 (2012). 22622582 10.1038/nature11051

[8] Yang, X., Purdue, P. E. & Lazarow, P. B. Eci1p uses a PTS1 to enter peroxisomes: either its own or that of a partner, Dci1p. *Eur. J. Cell. Biol.* **80**, 126–138 (2001). 11302517 10.1078/0171-9335-00144

[9] Islinger, M., Li, K. W., Seitz, J., Volkl, A. & Luers, G. H. Hitchhiking of cu/zn superoxide dismutase to peroxisomes–evidence for a natural piggyback import mechanism in mammals. *Traffic* **10**, 1711–1721. 10.1111/j.1600-0854.2009.00966.x (2009). 19686298 10.1111/j.1600-0854.2009.00966.x

[10] Reumann, S. Toward a definition of the complete proteome of plant peroxisomes: where experimental proteomics must be complemented by bioinformatics. *Proteomics* **11**, 1764–1779. 10.1002/pmic.201000681 (2011). 21472859 10.1002/pmic.201000681

[11] Huh, W. K. et al. Global analysis of protein localization in budding yeast. *Nature* **425**, 686–691. 10.1038/nature02026 (2003). 14562095 10.1038/nature02026

[12] Lingner, T. et al. Identification of novel plant peroxisomal targeting signals by a combination of machine learning methods and *in vivo* subcellular targeting analyses. *Plant. Cell.* **23**, 1556–1572. 10.1105/tpc.111.084095 (2011). 21487095 10.1105/tpc.111.084095 PMC3101550

[13] Reumann, S. et al. Proteome analysis of *Arabidopsis* leaf peroxisomes reveals novel targeting peptides, metabolic pathways, and defense mechanisms. *Plant. Cell.* **19**, 3170–3193. 10.1105/tpc.107.050989 (2007). 17951448 10.1105/tpc.107.050989 PMC2174697

[14] Reumann, S. et al. In-depth proteome analysis of *Arabidopsis* leaf peroxisomes combined with *in vivo* subcellular targeting verification indicates novel metabolic and regulatory functions of peroxisomes. *Plant. Physiol.* **150**, 125–143. 10.1104/pp.109.137703 (2009). 19329564 10.1104/pp.109.137703 PMC2675712

[15] Reumann, S., Chowdhary, G. & Lingner, T. Characterization, prediction and evolution of plant peroxisomal targeting signals type 1 (PTS1s). *Biochim. Biophys. Acta*. **1863**, 790–803. 10.1016/j.bbamcr.2016.01.001 (2016). 26772785 10.1016/j.bbamcr.2016.01.001

[16] Skoulding, N. S. et al. Experimental validation of plant peroxisomal targeting prediction algorithms by systematic comparison of in vivo import efficiency and in vitro PTS1 binding affinity. *J. Mol. Biol.* **427**, 1085–1101. 10.1016/j.jmb.2014.12.003 (2015). 25498386 10.1016/j.jmb.2014.12.003

[17] Kragler, F., Lametschwandtner, G., Christmann, J., Hartig, A. & Harada, J. J. Identification and analysis of the plant peroxisomal targeting signal 1 receptor NtPEX5. *Proc. Natl. Acad. Sci. U.S.A.* **95**, 13336–13341. 10.1073/pnas.95.22.13336 (1998). 9789089 10.1073/pnas.95.22.13336 PMC23804

[18] Lametschwandtner, G. et al. The difference in recognition of terminal tripeptides as peroxisomal targeting signal 1 between yeast and human is due to different affinities of their receptor Pex5p to the cognate signal and to residues adjacent to it. *J. Biol. Chem.* **273**, 33635–33643. 10.1074/jbc.273.50.33635 (1998). 9837948 10.1074/jbc.273.50.33635

[19] Reumann, S., Buchwald, D. & Lingner, T. PredPlantPTS1: A web server for the prediction of plant peroxisomal proteins. *Front. Plant. Sci.* **3**, 194. 10.3389/fpls.2012.00194 (2012). 22969783 10.3389/fpls.2012.00194 PMC3427985

[20] Williams, C., Bener Aksam, E., Gunkel, K., Veenhuis, M. & van der Klei, I. J. The relevance of the non-canonical PTS1 of peroxisomal catalase. *Biochim. Biophys. Acta*. **1823**, 1133–1141. 10.1016/j.bbamcr.2012.04.006 (2012). 22546606 10.1016/j.bbamcr.2012.04.006

[21] Magliano, P., Flipphi, M., Arpat, B. A., Delessert, S. & Poirier, Y. Contributions of the peroxisome and beta-oxidation cycle to biotin synthesis in fungi. *J. Biol. Chem.* **286**, 42133–42140. 10.1074/jbc.M111.279687 (2011). 21998305 10.1074/jbc.M111.279687 PMC3234907

[22] Bokor, E. et al. A complete nicotinate degradation pathway in the microbial eukaryote Aspergillus Nidulans. *Commun. Biol.* **5**, 723. 10.1038/s42003-022-03684-3 (2022). 35864155 10.1038/s42003-022-03684-3 PMC9304392

[23] Galanopoulou, K. et al. Purine utilization proteins in the eurotiales: cellular compartmentalization, phylogenetic conservation and divergence. *Fungal Genet. Biology: FG B*. **69**, 96–108. 10.1016/j.fgb.2014.06.005 (2014). 24970358 10.1016/j.fgb.2014.06.005

[24] Sprote, P., Brakhage, A. A. & Hynes, M. J. Contribution of peroxisomes to penicillin biosynthesis in *Aspergillus Nidulans*. *Eukaryot. Cell*. **8**, 421–423. 10.1128/EC.00374-08 (2009). 19151327 10.1128/EC.00374-08 PMC2653248

[25] Lu, Q. & McAlister-Henn, L. Peroxisomal localization and function of NADP+ -specific isocitrate dehydrogenases in yeast. *Arch. Biochem. Biophys.* **493**, 125–134. 10.1016/j.abb.2009.10.011 (2010). 19854152 10.1016/j.abb.2009.10.011 PMC2812674

[26] Stanley, W. A. & Wilmanns, M. Dynamic architecture of the peroxisomal import receptor Pex5p. *Biochim. Biophys. Acta*. **1763**, 1592–1598. 10.1016/j.bbamcr.2006.10.015 (2006). 17141887 10.1016/j.bbamcr.2006.10.015

[27] Flipphi, M., Oestreicher, N., Nicolas, V., Guitton, A. & Velot, C. The *Aspergillus Nidulans acul* gene encodes a mitochondrial carrier required for the utilization of carbon sources that are metabolized via the TCA cycle. *Fungal Genet. Biology: FG B*. **68**, 9–22. 10.1016/j.fgb.2014.04.012 (2014). 10.1016/j.fgb.2014.04.012 24835019

[28] Grigoriev, I. V. et al. Fueling the future with fungal genomics. *Mycology* **2**, 192–209. 10.1080/21501203.2011.584577 (2011).

[29] Grigoriev, I. V. et al. MycoCosm portal: gearing up for 1000 fungal genomes. *Nucleic Acids Res.* **42**, D699–704. 10.1093/nar/gkt1183 (2014). 24297253 10.1093/nar/gkt1183 PMC3965089

[30] Aranda, A. & Proudfoot, N. Transcriptional termination factors for RNA polymerase II in yeast. *Mol. Cell.* **7**, 1003–1011. 10.1016/s1097-2765(01)00235-0 (2001). 11389847 10.1016/s1097-2765(01)00235-0

[31] Zhang, Y. I-TASSER server for protein 3D structure prediction. *BMC Bioinform.* **9**, 40. 10.1186/1471-2105-9-40 (2008). 10.1186/1471-2105-9-40 PMC2245901 18215316

[32] Kelley, L. A., Mezulis, S., Yates, C. M., Wass, M. N. & Sternberg, M. J. The Phyre2 web portal for protein modeling, prediction and analysis. *Nat. Protoc.* **10**, 845–858. 10.1038/nprot.2015.053 (2015). 25950237 10.1038/nprot.2015.053 PMC5298202

[33] Amon, J. et al. A eukaryotic nicotinate-inducible gene cluster: convergent evolution in fungi and bacteria. *Open. Biol.* **7**, 170199. 10.1098/rsob.170199 (2017). 29212709 10.1098/rsob.170199 PMC5746545

[34] Bokor, E. et al. Genome organization and evolution of a eukaryotic nicotinate co-inducible pathway. *Open. Biol.* **11**, 210099. 10.1098/rsob.210099 (2021). 34582709 10.1098/rsob.210099 PMC8478523

[35] Sibthorp, C. et al. Transcriptome analysis of the filamentous fungus *Aspergillus Nidulans* directed to the global identification of promoters. *BMC Genom.* **14**, 847. 10.1186/1471-2164-14-847 (2013). 10.1186/1471-2164-14-847 PMC4046813 24299161

[36] Steyer, J. T., Downes, D. J., Hunter, C. C., Migeon, P. A. & Todd, R. B. Duplication and functional divergence of branched-chain amino acid biosynthesis genes in *Aspergillus nidulans*. *mBio* **12**, e0076821. 10.1128/mBio.00768-21 (2021). 10.1128/mBio.00768-21 PMC8262921 34154419

[37] Coradetti, S. T., Xiong, Y. & Glass, N. L. Analysis of a conserved cellulase transcriptional regulator reveals inducer-independent production of cellulolytic enzymes in *Neurospora crassa*. *Microbiologyopen* **2**, 595–609. 10.1002/mbo3.94 (2013). 23766336 10.1002/mbo3.94 PMC3948607

[38] Wang, F. et al. Transcription in fungal conidia before dormancy produces phenotypically variable conidia that maximize survival in different environments. *Nat. Microbiol.* **6**, 1066–1081. 10.1038/s41564-021-00922-y (2021). 34183813 10.1038/s41564-021-00922-y

[39] Kircher, S. et al. Light quality-dependent nuclear import of the plant photoreceptors phytochrome A and B. *Plant. Cell.* **11**, 1445–1456. 10.1105/tpc.11.8.1445 (1999). 10449579 10.1105/tpc.11.8.1445 PMC144301

[40] Ast, J., Stiebler, A. C., Freitag, J. & Bolker, M. Dual targeting of peroxisomal proteins. *Front. Physiol.* **4**, 297. 10.3389/fphys.2013.00297 (2013). 24151469 10.3389/fphys.2013.00297 PMC3798809

[41] Kragler, F., Langeder, A., Raupachova, J., Binder, M. & Hartig, A. Two independent peroxisomal targeting signals in catalase A of *Saccharomyces cerevisiae*. *J. Cell Biol.* **120**, 665–673. 10.1083/jcb.120.3.665 (1993). 8425895 10.1083/jcb.120.3.665 PMC2119545

[42] Elgersma, Y. et al. Analysis of the carboxyl-terminal peroxisomal targeting signal 1 in a homologous context in *Saccharomyces cerevisiae*. *J. Biol. Chem.* **271**, 26375–26382. 10.1074/jbc.271.42.26375 (1996). 8824293 10.1074/jbc.271.42.26375

[43] de Alves, P. et al. Aspergillus fumigatus mitogen-activated protein kinase MpkA is involved in gliotoxin production and self-protection. *Nat. Commun.* **15**, 33. 10.1038/s41467-023-44329-1 (2024). 38167253 10.1038/s41467-023-44329-1 PMC10762094

[44] Khan, Z., Michalopoulos, G. K. & Stolz, D. B. Peroxisomal localization of hypoxia-inducible factors and hypoxia-inducible factor regulatory hydroxylases in primary rat hepatocytes exposed to hypoxia-reoxygenation. *Am. J. Pathol.* **169**, 1251–1269. 10.2353/ajpath.2006.060360 (2006). 17003483 10.2353/ajpath.2006.060360 PMC1698853

[45] Valette-Collet, O., Cimerman, A., Reignault, P., Levis, C. & Boccara, M. Disruption of *Botrytis cinerea* pectin methylesterase gene Bc*pme1* reduces virulence on several host plants. *Mol. Plant. Microbe Interact.* **16**, 360–367. 10.1094/MPMI.2003.16.4.360 (2003). 12744465 10.1094/MPMI.2003.16.4.360

[46] Sella, L. et al. Involvement of fungal pectin methylesterase activity in the interaction between *Fusarium graminearum* and wheat. *Mol. Plant. Microbe Interact.* **29**, 258–267. 10.1094/MPMI-07-15-0174-R (2016). 26713352 10.1094/MPMI-07-15-0174-R

[47] Rocha, V. D. D., Dal’Sasso, T., Dal-Bianco, M. & Oliveira, L. O. Genome-wide survey and evolutionary history of the pectin methylesterase (PME) gene family in the *Dothideomycetes* class of fungi. *Fungal Genet. Biology: FG B*. **169**, 103841. 10.1016/j.fgb.2023.103841 (2023). 10.1016/j.fgb.2023.103841 37797717

[48] Mingora, C., Ewer, J. & Ospina-Giraldo, M. Comparative structural and functional analysis of genes encoding pectin methylesterases in *Phytophthora spp*. *Gene* **538**, 74–83. 10.1016/j.gene.2014.01.016 (2014). 24434809 10.1016/j.gene.2014.01.016

[49] Zhao, Z., Liu, H., Wang, C. & Xu, J. R. Comparative analysis of fungal genomes reveals different plant cell wall degrading capacity in fungi. *BMC Genom.* **14**, 274. 10.1186/1471-2164-14-274 (2013). 10.1186/1471-2164-14-274 PMC3652786 23617724

[50] Karacsony, Z., Gacser, A., Vagvolgyi, C., Scazzocchio, C. & Hamari, Z. A dually located multi-HMG-box protein of *Aspergillus Nidulans* has a crucial role in conidial and ascospore germination. *Mol. Microbiol.* **94**, 383–402. 10.1111/mmi.12772 (2014). 25156107 10.1111/mmi.12772

[51] Yanisch-Perron, C., Vieira, J. & Messing, J. Improved M13 phage cloning vectors and host strains: nucleotide sequences of the M13mp18 and pUC19 vectors. *Gene* **33**, 103–119. 10.1016/0378-1119(85)90120-9 (1985). 2985470 10.1016/0378-1119(85)90120-9

[52] Hanahan, D. Studies on transformation of *Escherichia coli* with plasmids. *J. Mol. Biol.* **166**, 557–580. 10.1016/s0022-2836(83)80284-8 (1983). 6345791 10.1016/s0022-2836(83)80284-8

[53] Sambrook, J., Fritsch, E. F. & Maniatis, T. *Molecular Cloning: A Laboratory Manual* (Cold Spring Harbor Laboratory Press, 1989).

[54] Yu, J. H. et al. Double-joint PCR: a PCR-based molecular tool for gene manipulations in filamentous fungi. *Fungal Genet. Biology: FG B*. **41**, 973–981. 10.1016/j.fgb.2004.08.001 (2004). 10.1016/j.fgb.2004.08.001 15465386

[55] Specht, C. A., DiRusso, C. C., Novotny, C. P. & Ullrich, R. C. A method for extracting high-molecular-weight deoxyribonucleic acid from fungi. *Anal. Biochem.* **119**, 158–163. 10.1016/0003-2697(82)90680-7 (1982). 7041693 10.1016/0003-2697(82)90680-7

[56] Larionov, A., Krause, A. & Miller, W. A standard curve based method for relative real time PCR data processing. *BMC Bioinform.* **6**, 62. 10.1186/1471-2105-6-62 (2005). 10.1186/1471-2105-6-62 PMC1274258 15780134

[57] Ferenczy, L., Kevei, F. & Szegedi, M. Increased fusion frequency of *Aspergillus Nidulans* protoplasts. *Experientia* **31**, 50–52. 10.1007/BF01924674 (1975). 1089544 10.1007/BF01924674

[58] Kevei, F. & Peberdy, J. F. Interspecific hybridization between *Aspergillus Nidulans* and *Aspergillus rugulosus* by fusion of somatic protoplasts. *J. Gen. Microbiol.* **102**, 255–262. 10.1099/00221287-102-2-255 (1977).

[59] Antal, Z., Manczinger, L. & Ferenczy, L. Transformation of a mycoparasitic *Trichoderma Harzianum* strain with the *ArgB* gene of *Aspergillus Nidulans*. *Biotechnol. Tech.* **11**, 205–208. 10.1023/A:1018417917255 (1997).

[60] Bradford, M. M. A rapid and sensitive method for the quantitation of microgram quantities of protein utilizing the principle of protein-dye binding. *Anal. Biochem.* **72**, 248–254 (1976). 942051 10.1016/0003-2697(76)90527-3

[61] Laemmli, U. K. Cleavage of structural proteins during the assembly of the head of bacteriophage T4. *Nature* **227**, 680–685 (1970). 5432063 10.1038/227680a0

[62] Thumuluri, V., Almagro Armenteros, J. J., Johansen, A. R., Nielsen, H. & Winther, O. DeepLoc 2.0: multi-label subcellular localization prediction using protein Language models. *Nucleic Acids Res.*10.1093/nar/gkac278 (2022). 10.1093/nar/gkac278 PMC9252801 35489069

[63] Steinegger, M. & Soding, J. MMseqs2 enables sensitive protein sequence searching for the analysis of massive data sets. *Nat. Biotechnol.* **35**, 1026–1028. 10.1038/nbt.3988 (2017). 29035372 10.1038/nbt.3988

[64] Paysan-Lafosse, T. et al. InterPro in 2022. *Nucleic Acids Res.* **51**, D418–D427. 10.1093/nar/gkac993 (2023). 36350672 10.1093/nar/gkac993 PMC9825450

[65] Honorato, R. V. et al. Structural biology in the clouds: the WeNMR-EOSC ecosystem. *Front. Mol. Biosci.* **8**, 729513. 10.3389/fmolb.2021.729513 (2021). 34395534 10.3389/fmolb.2021.729513 PMC8356364

[66] Honorato, R. V. et al. The HADDOCK2.4 web server for integrative modeling of biomolecular complexes. *Nat. Protoc.* **19**, 3219–3241. 10.1038/s41596-024-01011-0 (2024). 38886530 10.1038/s41596-024-01011-0

