# Supplementary material for: A non-canonical fungal peroxisome PTS-1 signal, SYM, and its evolutionary aspects
Source: Sci Rep. 2025 Aug 1;15:28088. doi: 10.1038/s41598-025-13871-x (PMC12316961; doi:10.1038/s41598-025-13871-x)
Supplement: Supplementary file 2 — Supplementary Material 2 [file 41598_2025_13871_MOESM2_ESM.pdf]

SUPPLEMENTARY FIGURES

for

A non-canonical fungal peroxisome PTS-1 signal, SYM, and its evolutionary aspects

Judit Ámon[1#], Suren Nemuuzaya[#1], Kevin Alczheimer[1], Sándor Kocsubé[1], Zoltán Farkas[2], Gergő Svorenj[3], Attila Gácser[1,3], Chetna Tyagi[1], László Kozma-Bognár[4,5] and Zsuzsanna Hamari[1]*

[1]University of Szeged Faculty of Science and Informatics, Department of Biotechnology and Microbiology, Szeged, Hungary

[2]Synthetic and Systems Biology Unit, Institute of Biochemistry, HUN-REN Biological Research Centre, Szeged, National Laboratory of Biotechnology HU-6726, Hungary

[3]HCEMM-USZ Fungal Pathogens Research Group, Department of Microbiology, Faculty of Science and Informatics, University of Szeged, Szeged, Hungary

[4]University of Szeged Faculty of Science and Informatics, Department of Genetics, Szeged, Hungary

[5]Institute of Plant Biology, Biological Research Centre, Hungarian Research Network (HUN-REN), Szeged, Hungary

[#] These authors contributed equally

* Corresponding author: hamari@bio.u-szeged.hu,

Content:

Supplementary Fig. S1: Interaction analysis between Pex5 receptors and various cargo proteins.

Supplementary Fig. S2: Detection of Gfp and Gfp-tagged fusion proteins in strains used in experiments for Figure 1 and Figure 6.

Supplementary Fig. S3: Detailed distribution of SYM-proteins in fungi.

Supplementary Fig. S4: Intracellular locations of SYM proteins across fungal genera.

Supplementary Fig. S5: Clusters with multiple representing genera.

Supplementary Fig. S6: Uncropped images of Western blots shown in Supplementary Fig. S2.

(*a*)

Binding energies (kcal/mol) of non-tagged and PTS-1 tagged Gfp proteins with plant, human and fungal Pex5-TPR domains.

	Gfp	Gfp-**SKL** **(C_{-3} C_{-2} C_{-1})**	Gfp-**SRL** **(C_{-3} C_{-2} C_{-1})**	Gfp-**AKL** **(C_{-3} C_{-2} C_{-1})**	Gfp-**SYM** **(C_{-3}** NC_{-2} **C_{-1})**	Gfp-**SYI** **(C_{-3}** NC_{-2} **C_{-1})**	Gfp-**SYL** **(C_{-3}** NC_{-2} **C_{-1})**	Gfp-**SYF** **(C_{-3}** NC_{-2} NC_{-1}**)**	Gfp-**SYY** **(C_{-3}** NC_{-2} NC_{-1}**)**	Gfp-**SYV** **(C_{-3}** NC_{-2} NC_{-1}**)**
A. thaliana	-75.9 +/- 6.5	-113.2 +/- 7.9	-83.0 +/- 3.6	-88.7 +/- 4.0	-75.1 +/- 4.6	-80.8 +/- 7.9	-73.1 +/- 6.4	-124.9 +/- 2.3	-102.1 +/- 2.3	-85.4 +/- 2.0
H. sapiens only TPR	-74.1 +/- 6.9	-90.0 +/- 0.5	-87.4 +/- 10.3	-89.6 +/- 1.7	-80.9 +/- 3.4	-72.0 +/- 5.3	-77.2 +/- 2.7	-96.3 +/- 0.7	-94.5 +/- 6.3	-83.2 +/- 5.2
A. nidulans only TPR	-72.5 +/- 5.6	-88.5 +/- 1.4	-97.6 +/- 4.2	-91.2 +/- 7.2*	-78.5 +/- 1.0	-72.0 +/- 1.4	-71.3 +/- 2.6	-86.6 +/- 3.0	-95.9 +/- 5.7	-73.8 +/- 7.7
S. cerevisiae only TPR	-73.1 +/- 2.5	-101.4 +/- 1.6	-76.5 +/- 0.6	-114.0 +/- 4.5	-86.0 +/- 4.8	-88.7 +/- 9.5	-89.0 +/- 20.5	-97.9 +/- 1.9	-87.2 +/- 4.5	-81.6 +/- 10.8

* In case of canonical AKL motif, terminal decapeptide of Gfp-AKL does not directly enter the cavity formed by the TPR domain of *A. nidulans* PexE, but turns side-ways and binding involves upstream residues like Lys239, Glu236, Met234, and Gly233, which establish strong interactions. To find out, whether natural, reported peroxisomal cargo proteins with -AKL signal enters the TPR cavity, we modelled the binding of FoxA and SidF proteins to PexE. Scores for FoxA (peroxisomal hydratase-dehydrogenase-epimerase) and SidF (peroxisomal triacetylfusarinine C biosynthetic transacylase) (-100.7 +/- 12.4 and -132.9 +/- 8.7 kcal/mol, respectively) reflect high interactions with PexE, and importantly, the interaction occurred outside of the TPR cavity, similarly as it was observed for AKL-tagged Gfp. Therefore, these data suggest that the canonical AKL signal in *A. nidulans* is stabilized by the upstream residues and does not interact in the same manner as it does with the human Pex5.

(***b***)

>Gfp

```
MVSKGEELFTGVVPILVELDGDVNGHKFSVSGEGEGDATYGKLTLKFICTTGKLPVPWPTLVTTFTYGVQCFSRY
PDHMKQHDFFKSAMPEGYVQERTIFFKDDGNYKTRAEVKFEGDTLVNRIELKGIDFKEDGNILGHKLEYNYNSHN
VYIMADKQKNGIKVNFKIRHNIEDGSVQLADHYQQNTPIGDGPVLLPDNHYLSTQSALSKDPNEKRDHMVLLEFV
TAAGITHGMDELYK
```

>PexE

```
MSFLGGAECSTAGNPLTQFTKHVQDDKSLQRDRLVGRAPGMQEGMRSQGMMGGHDQMMDEFAQQSAQLPGGPQQH
MRMEMEQVRQQLEQMHTTPRTGSPGWAAEFDPGEQARMEAAFAGPKGPMMNNGSGFTPAEFARFQQQSTMSVPQS
ASPVTAGQSPMMGGYQRSMGMGYMGYGGMGMMQPGFGPMGMQHQQPAEASTQDKGKGRMIELDDENWEAQFKEIE
TADQGKLDDEANAAIEAELNDLDRSVPTTSSTEDLSHFERVWERVQAETATNRKLAEDSEYNIDDNLHMGDMAEW
DGFDNLNTRFREPRLGDYSFEQENVFRDIANPFEEGMKIMQEGGNLSLAALAFEAAVQKDPQHVKAWTMLGTAQA
QNEKELPAIRALEQALKVDPNNLDALMGLAVSYTNEGYDSTAYRTLERWLSVKYPQIISRDDLSSDADLGFTDRQ
ILHERVTDLFIQAAQLSPSGAQMDPDVQVGLGVLFYCAEEYEKAVDCFTTALASTESGTTNQREQLHLLWNRLGA
TLANSGRSEEAIEAYEQALNINPNFVRARYNLGVSCINIGCYPEAAQHLLGALSMHRVVEEEGKERAREIVGGND
GRINEAELNRMITANQSTNLYDTLRRVFSQMGRRDLADLVEAGMDVNIFRKEFEF
```

(*c*)

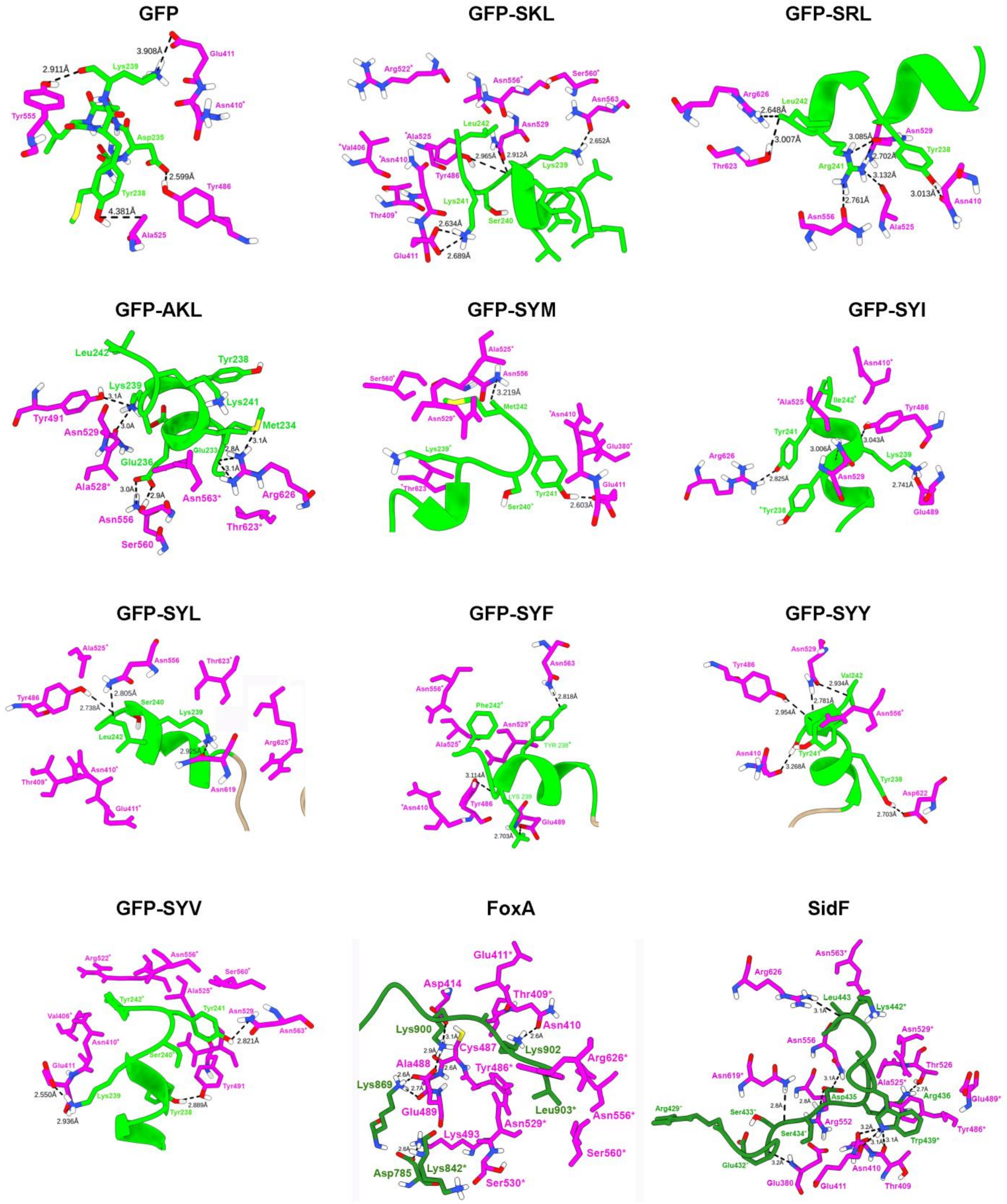

Supplementary Fig. S1. Interaction analysis between Pex5 receptors and various cargo proteins. (**a**) Binding energies (kcal/mol) of non-tagged and PTS-1 tagged Gfp proteins with Pex5 homologs from *Arabidopsis thaliana* (Q9FMA3), Homo sapiens (PDB code: 2C0L), *Aspergillus nidulans* (PexE, AN10215) and *Saccharomyces cerevisiae* (YDR244W) was calculated by using HADDOCK 2.4 server (for detailed results see Supplementary Data sheet

'HADDOCK results'). The canonical or non-canonical nature of the residues composing C-terminal tripeptide is indicated by "C" (canonical) or "NC" (non-canonical), shown in parentheses below each cargo protein name. (**b**) AA sequences of Gfp and PexE used for the modeled interactions shown in panel (c). The TPR domain is highlighted in grey. (**c**) Residues mediating the interaction between *A. nidulans* Pex5 and various Gfp-derived cargos, as well as two natural peroxisomal cargo proteins from *A. nidulans*: FoxA (AN7111, peroxisomal hydratase-dehydrogenase-epimerase) and SidF (AN6234, peroxisomal triacetylfusarinine C biosynthetic transacylase). PexE is shown in magenta, cargo proteins are shown in green. Hydrogen bonds are indicated with dashed lines, and distances between interacting atoms are given in Ångströms. Asterisks denote van der Waals or other secondary interactions.

Interpretation of the analysis is the following:

Interaction scores received from HADDOCK 2.4 binding simulation analysis reflect the free energy of binding (in kcal/mol); thus, more negative values indicate stronger interaction potential. As expected, the absence of a PTS-1 signal (first column in Fig. S1a) causes the lowest interaction with Pex5 (named PexE in *A. nidulans*).

Fully canonical signals such as SKL and SRL exhibit strong interaction scores across all species, highlighting the importance of a positively charged residue (K or R) at the C-terminal position (Fig. S1a). A minor exception is the slightly lower score observed for *S. cerevisiae* Pex5 with -SRL, potentially due to variation in the number and strength of Hydrogen bonds and other stabilizing interactions. For instance, in the *A. nidulans* PexE-Gfp-SKL complex, Lys241 forms two Hydrogen bonds with Glu411 in the TPR domain, while upstream Lys239 (at position -4) contributes three additional Hydrogen bonds with Tyr486, Asn529, and Asn563 (Fig. S1c). Similarly, in the *A. nidulans* PexE-Gfp-SRL complex, Arg241 and Leu242 engage in seven Hydrogen bonds with Ala525, Asn529, Asn556, Thr623, and Arg626 (Fig. S1c). Notably, Tyr238 (at position -5) also forms a Hydrogen bond with Asn410, contributing to the high binding affinity (Fig. S1c).

Replacing a single canonical signal residue to non-canonical residue (as in the SYM, SYI, and SYL signals) leads to reduced interaction affinity with *A. nidulans* PexE, as reflected in their respective scores (Fig. S1a). These motifs retain only one aromatic residue. However, interaction strength is markedly restored or even enhanced in SYF and SYY variants, despite also containing only one canonical residue (Fig. S1a). In these cases, upstream residues Lys239 (at position -4) and Tyr238 (at position -5) form compensatory Hydrogen and ionic

bonds with Pex5 residues such as Tyr486, Glu489, and Asn563 (Fig. S1c). This suggests that loss of a canonical PTS-1 motif can be functionally mitigated by upstream residues, which establish alternative stabilizing interactions within the TPR cavity. The high interaction score of SYY is further supported by multiple van der Waals contacts (Fig. S1c).

Conversely, although AKL-tagged Gfp shows strong binding to human Pex5, initial docking simulations with *A. nidulans* PexE yielded unexpectedly low scores (~ -64 kcal/mol) when the C-terminal tail entered the TPR cavity in the same orientation as in the human complex. When simulations were repeated with increased flexibility in the C-terminal 15 residues, the score improved to -91.2 ± 7.2 kcal/mol (Fig. S1a). This alternative conformation involved a lateral entry of the C-terminal tail, with upstream residues such as Lys239, Glu236, Met234, and Gly233 participating in key interactions (Fig. S1c). To test whether this interaction mode is biologically relevant, we analyzed two natural AKL-containing cargo proteins, FoxA (AN7111, peroxisomal hydratase-dehydrogenase-epimerase) and SidF (AN6234, peroxisomal triacetylfusarinine C biosynthetic transacylase), both known to bind *A. nidulans* PexE *in vivo*. Their docking simulations showed high binding scores (-100.7 ± 12.4 and -132.9 ± 8.7 kcal/mol, respectively), with similar lateral tail conformations and involvement of upstream residues (Fig. S1c). These results suggest that the AKL signal does not bind *A. nidulans* PexE in the same manner as it does human Pex5, and its stabilization likely depends on extended upstream contacts.

Additionally, a weak interaction was observed between *A. thaliana* Pex5 and Gfp-SYL, where the terminal tripeptide engages only in van der Waals interactions with the TPR domain (Fig. S1a and S1c). In *A. nidulans* PexE, only Leu242 forms a hydrogen bond (with Asn556), while the other residues contribute minimal contacts (Fig. S1c). In contrast, binding improves in the human Pex5 complex, where Ser240 and Tyr241 form hydrogen bonds with Asn561 and Ser565, respectively, and Lys239 engages in an ionic interaction with Glu416 (Fig. S1c).

In conclusion, canonical PTS-1 tripeptides confer strong binding to Pex5 homologs across species (Fig. S1a). Loss of one canonical residue reduces binding affinity but can be partially compensated by upstream interactions (Fig. S1a and S1c). When two canonical residues are lost, the interaction depends strongly on the nature of the substitutions: aromatic replacements (e.g., SYF, SYY) may enhance binding due to additional stabilizing contacts involving upstream residues (Fig. S1a and S1c). Notably, Lys239 (at position -4) and Tyr238 (at position -5) in the tagged Gfp variants (corresponding to the last two C-terminal residues (positions -1 and -2) in the non-tagged GFP protein (Fig. S1b)) emerge as key contributors in stabilizing these non-canonical signals. Finally, the concave geometry of the TPR domain surface also appears to influence the conformation and binding mode of C-terminal targeting signals, as exemplified by the -AKL motif’s interaction with *A. nidulans* PexE and human Pex5.

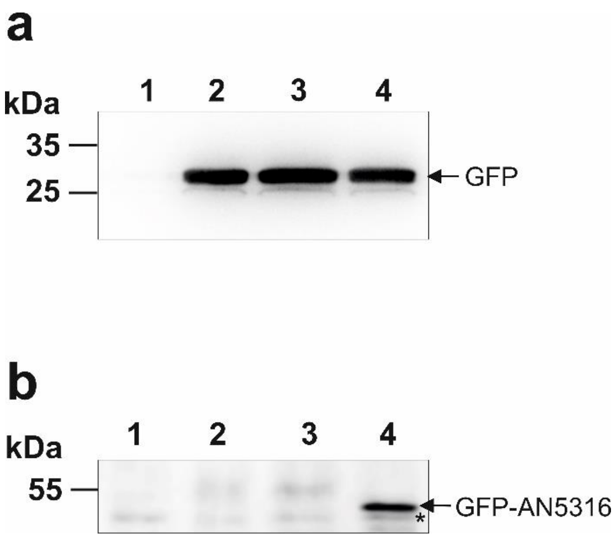

Supplementary Fig. S2. Supplementary Fig. S2. Detection of Gfp and Gfp-tagged fusion proteins in strains used in experiments for Figure 1 and Figure 6. Uncropped images of Western blots are shown in Supplementary Fig. S6.

(**a**) Lane 1: non-transformed control (strain HZS.573), lane 2: Gfp (strain HZS.948), lane 3: Gfp-SRL (strain HZS.952), lane 4: Gfp-SYM (HZS.949). These strains were used for fluorescence microscopy shown in Figure 1b. The arrow indicates the position of Gfp and its derivatives.

(**b**) Lane 1: non-transformed control (strain HZS.573), lane 2: Gfp-SRL (strain HZS.952), lane 3: Gfp-SYM (HZS.949), lane 4: Gfp-AN5316 (HZS.1041). Strain HZS.1041 was used for fluorescence microscopy shown in Figure 6. The arrow points to the position of the Gfp-AN5316 fusion protein. The asterisk indicates a non-specific band present in all samples.

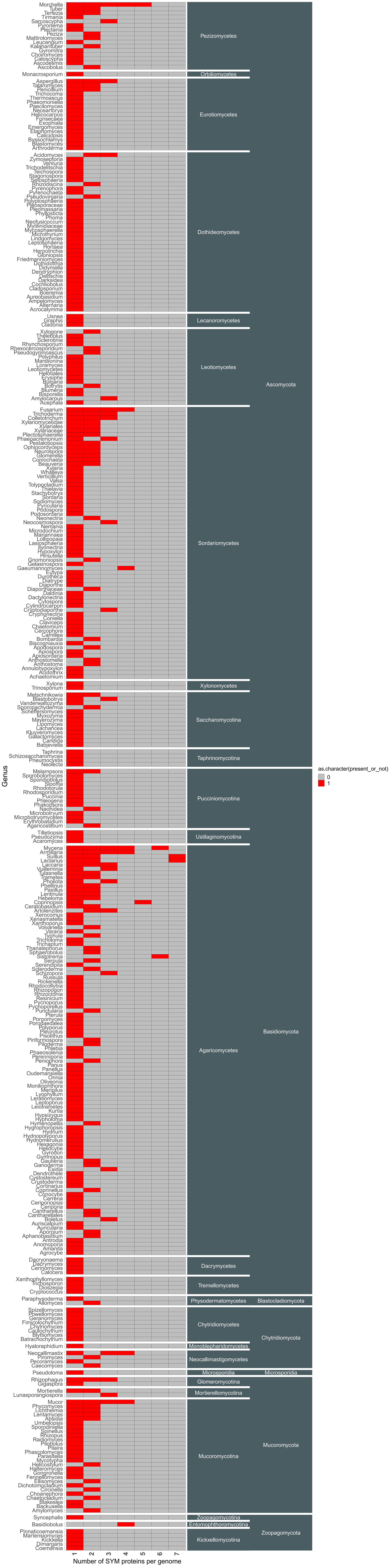

Genus
Number of SYM proteins per genome
1
2
3
4
5
6
7
as.character(present_or_not)
0
1
Pezizomycetes
Orbiliomycetes
Eurotiomycetes
Dothideomycetes
Lecanoromycetes
Leotiomycetes
Sordariomycetes
Xylonomycetes
Saccharomycotina
Taphrinomycotina
Ascomycota
Pucciniomycotina
Ustilaginomycotina
Agaricomycetes
Dacrymycetes
Tremellomycetes
Basidiomycota
Physodermatomycetes
Blastocladiomycota
Chytridiomycetes
Monoblepharidomycetes
Neocallimastigomycetes
Chytridiomycota
Microsporidia
Microsporidia
Glomeromycotina
Mortierellomycotina
Mucoromycotina
Mucoromycota
Zoopagomycotina
Entomophthoromycotina
Kickxellomycotina
Zoopagomycota

Supplementary Fig. S3. Detailed distribution of SYM-proteins in fungi. The heatmap shows the number of SYM proteins per genome across fungal genera. Columns represent the number of SYM-proteins per genome; rows represent various genera; red indicates the presence of a given number of SYM proteins within a taxonomic unit. Data is available in Supplementary Data sheet 'Fig_S3_sym_heatmap_detailed'.

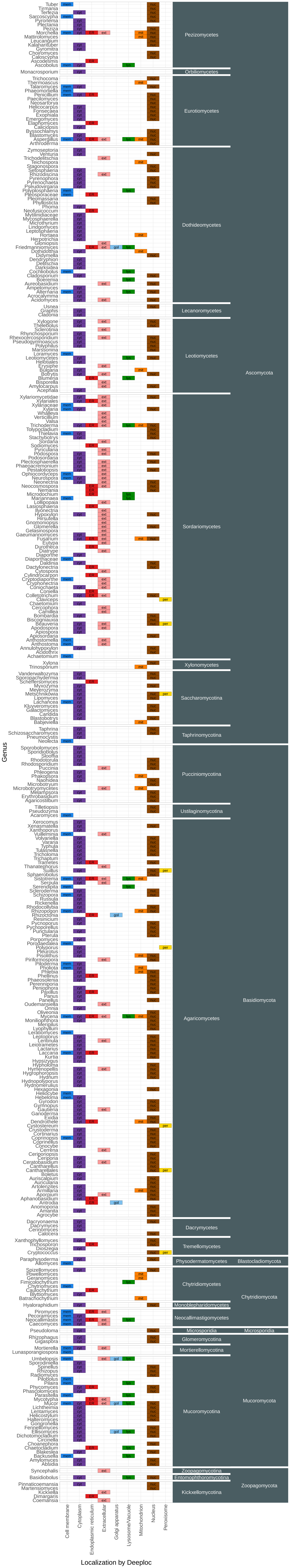
Genus
Localization by Deeploc
Cell membrane
Cytoplasm
Endoplasmic reticulum
Extracellular
Golgi apparatus
Lysosome/Vacuole
Mitochondrion
Nucleus
Peroxisome
Pezizomycetes
Orbiliomycetes
Eurotiomycetes
Dothideomycetes
Lecanoromycetes
Leotiomycetes
Ascomycota
Sordariomycetes
Xylonomycetes
Saccharomycotina
Taphrinomycotina
Pucciniomycotina
Ustilaginomycotina
Agaricomycetes
Basidiomycota
Dacrymycetes
Tremellomycetes
Physodermatomycetes
Blastocladiomycota
Chytridiomycetes
Chytridiomycota
Monoblepharidomycetes
Neocallimastigomycetes
Microsporidia
Glomeromycotina
Mortierellomycotina
Mucoromycotina
Mucoromycota
Zoopagomycotina
Entomophthoromycotina
Kickxellomycotina
Zoopagomycota

Supplementary Fig. S4. Intracellular locations of SYM proteins across fungal genera. The heatmap shows the intracellular location (rows) of investigated SYM proteins across fungal genera (columns) belonging to various taxonomic units (right panels, class and phylum). For visualization purposes, the color of the heatmap also denotes the intracellular location, as predicted by DeepLock 2.0. Data is available in Supplementary Data sheet 'Fig_S4_detailed_deeploc'.

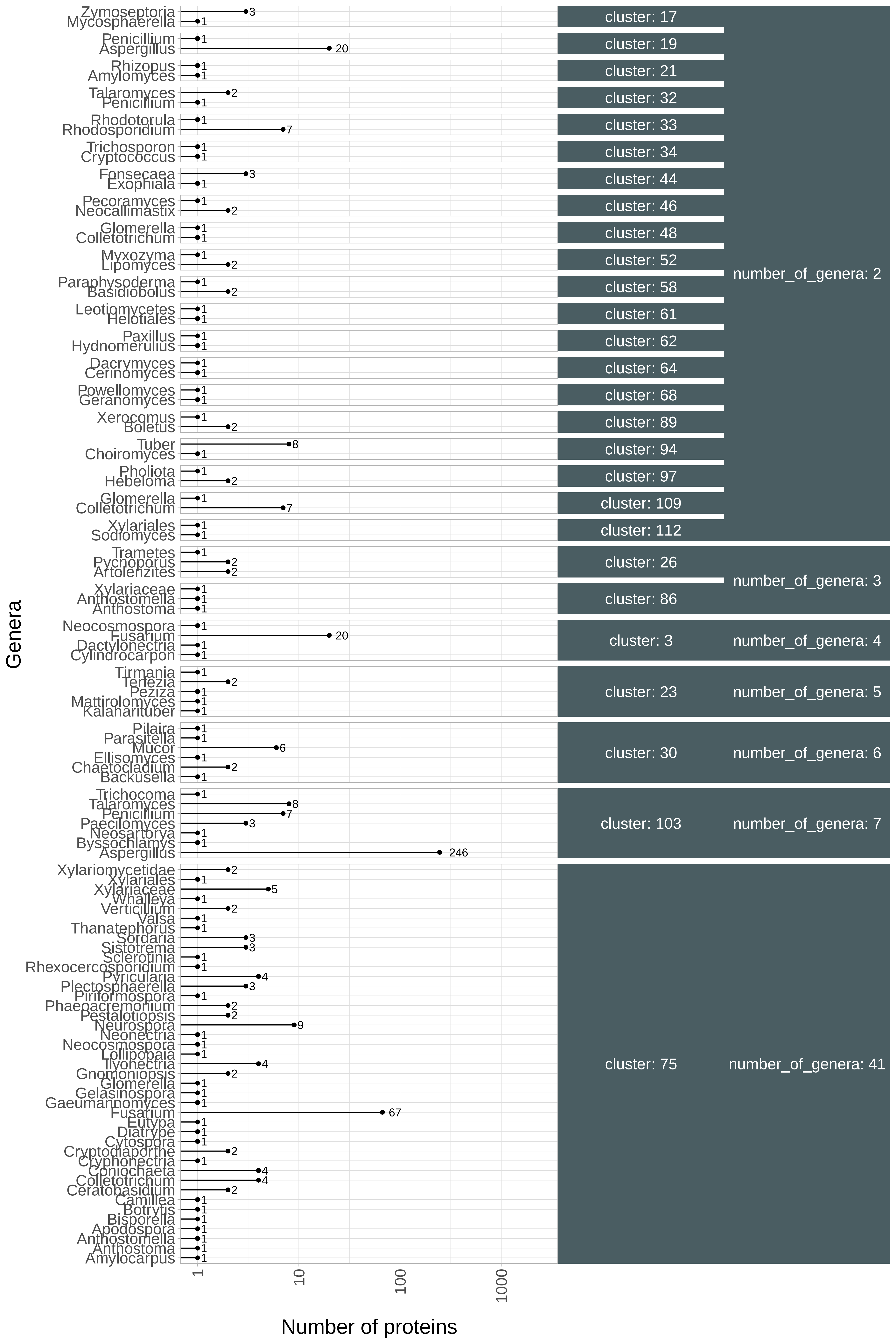

Genera
Number of proteins
1
10
100
1000
cluster: 17
cluster: 19
cluster: 21
cluster: 32
cluster: 33
cluster: 34
cluster: 44
cluster: 46
cluster: 48
cluster: 52
cluster: 58
cluster: 61
cluster: 62
cluster: 64
cluster: 68
cluster: 89
cluster: 94
cluster: 97
cluster: 109
cluster: 112
number_of_genera: 2
cluster: 26
cluster: 86
number_of_genera: 3
cluster: 3
number_of_genera: 4
cluster: 23
number_of_genera: 5
cluster: 30
number_of_genera: 6
cluster: 103
number_of_genera: 7
cluster: 75
number_of_genera: 41
Zymoseptoria 3
Mycosphaerella 1
Penicillium 1
Aspergillus 20
Rhizopus 1
Amylomyces 1
Talaromyces 2
Penicillium 1
Rhodotorula 1
Rhodosporidium 7
Trichosporon 1
Cryptococcus 1
Fonsecaea 3
Exophiala 1
Pecoramyces 1
Neocallimastix 2
Glomerella 1
Colletotrichum 1
Myxozyma 1
Lipomyces 2
Paraphysoderma 1
Basidiobolus 2
Leotiomycetes 1
Helotiales 1
Paxillus 1
Hydnomerulius 1
Dacrymyces 1
Cerinomyces 1
Powellomyces 1
Geranomyces 1
Xerocomus 1
Boletus 2
Tuber 8
Choiromyces 1
Pholiota 1
Hebeloma 2
Glomerella 1
Colletotrichum 7
Xylariales 1
Sodiomyces 1
Trametes 1
Pycnoporus 2
Artolenzites 2
Xylariaceae 1
Anthostomella 1
Anthostoma 1
Neocosmospora 1
Fusarium 20
Dactylonectria 1
Cylindrocarpon 1
Tirmania 1
Terfezia 2
Peziza 1
Mattirolomyces 1
Kalaharituber 1
Pilaira 1
Parasitella 1
Mucor 6
Ellisomyces 1
Chaetocladium 2
Backusella 1
Trichocoma 1
Talaromyces 8
Penicillium 7
Paecilomyces 3
Neosartorya 1
Byssochlamys 1
Aspergillus 246
Xylariomycetidae 2
Xylariales 1
Xylariaceae 5
Whalleya 1
Verticillium 2
Valsa 1
Thanatephorus 1
Sordaria 3
Sistotrema 3
Sclerotinia 1
Rhexocercosporidium 1
Pyricularia 4
Plectosphaerella 3
Piriformospora 1
Phaeoacremonium 2
Pestalotiopsis 2
Neurospora 9
Neonectria 1
Neocosmospora 1
Lollipopaia 1
Ilyonectria 4
Gnomoniopsis 2
Glomerella 1
Gelasinospora 1
Gaeumannomyces 1
Fusarium 67
Eutypa 1
Diatrype 1
Cytospora 1
Cryptodiaporthe 2
Cryphonectria 1
Coniochaeta 4
Colletotrichum 4
Ceratobasidium 2
Camillea 1
Botrytis 1
Bisporella 1
Apodospora 1
Anthostomella 1
Anthostoma 1
Amylocarpus 1

Supplementary Fig. S5. Clusters with multiple representing genera. The figure shows the number of SYM-proteins belonging to different clusters (middle panels) across genera. Clusters are grouped based on the number of representing genera (right panels). Only clusters that are composed of SYM-proteins from more than one genus is shown. Please note that a single genus can contain SYM-proteins belonging to multiple clusters. Data is available in Supplementary Data sheet 'Fig_S5_clusters_with_min2genera'.

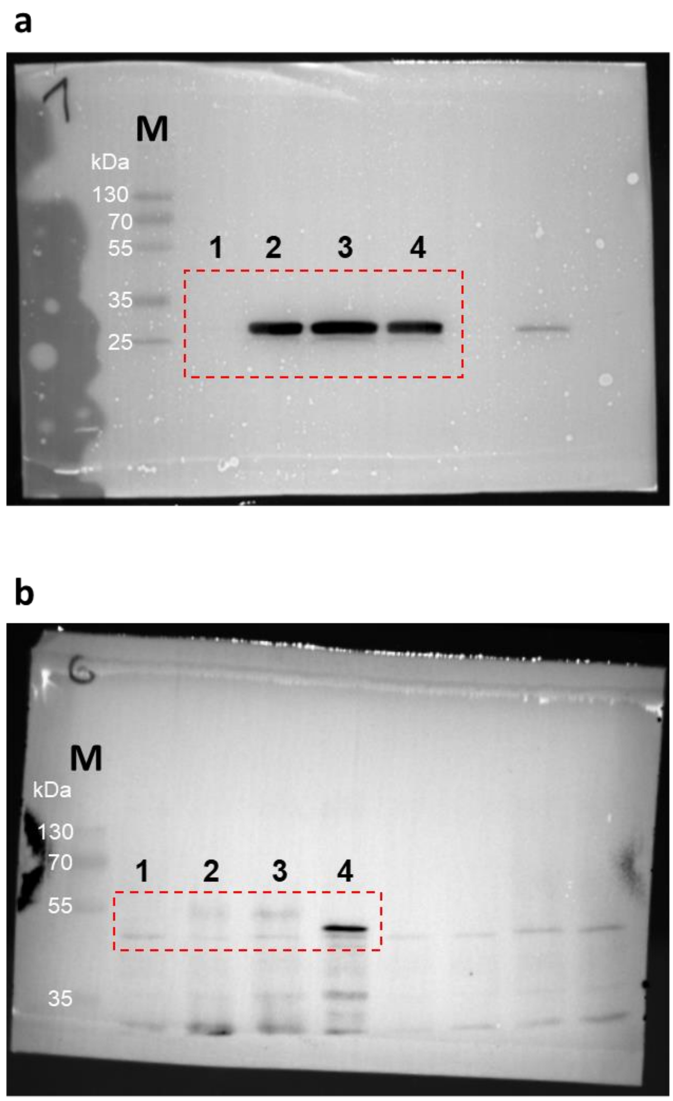

Supplementary Fig. S6. Uncropped images of Western blots shown in Supplementary Fig. S2.

(**a**) Lane 1: non-transformed control (strain HZS.573), lane 2: Gfp (strain HZS.948), lane 3: Gfp-SRL (strain HZS.952), lane 4: Gfp-SYM (HZS.949). These strains were used for fluorescence microscopy shown in Figure 1b.

(**b**) Lane 1: non-transformed control (strain HZS.573), lane 2: Gfp-SRL (strain HZS.952), lane 3: Gfp-SYM (HZS.949), lane 4: Gfp-AN5316 (HZS.1041). Strain HZS.1041 was used for fluorescence microscopy shown in Figure 6a.

Overlays of membrane images and chemiluminescens signals are shown. The sections that were cut for presentation in Supplementary Fig. S2 are indicated by rectangles with dashed red outline. Molecular size of the visible bands of PageRuler Plus protein marker is shown on the left side of the blots.
